# Biosynthesis of anticancer phytochemical compounds and their chemistry

**DOI:** 10.3389/fphar.2023.1136779

**Published:** 2023-03-09

**Authors:** Amandeep Dogra, Jitender Kumar

**Affiliations:** Department of Plant Science, School of Life Sciences, Central University of Himachal Pradesh, Dharamshala, India

**Keywords:** biosynthetic pathway, molecular docking, nanotecehnology, genetic engineering, phytochemical compounds

## Abstract

Cancer is a severe health issue, and cancer cases are rising yearly. New anticancer drugs have been developed as our understanding of the molecular mechanisms behind diverse solid tumors, and metastatic malignancies have increased. Plant-derived phytochemical compounds target different oncogenes, tumor suppressor genes, protein channels, immune cells, protein channels, and pumps, which have attracted much attention for treating cancer in preclinical studies. Despite the anticancer capabilities of these phytochemical compounds, systemic toxicity, medication resistance, and limited absorption remain more significant obstacles in clinical trials. Therefore, drug combinations of new phytochemical compounds, phytonanomedicine, semi-synthetic, and synthetic analogs should be considered to supplement the existing cancer therapies. It is also crucial to consider different strategies for increased production of phytochemical bioactive substances. The primary goal of this review is to highlight several bioactive anticancer phytochemical compounds found in plants, preclinical research, their synthetic and semi-synthetic analogs, and clinical trials. Additionally, biotechnological and metabolic engineering strategies are explored to enhance the production of bioactive phytochemical compounds. Ligands and their interactions with their putative targets are also explored through molecular docking studies. Therefore, emphasis is given to gathering comprehensive data regarding modern biotechnology, metabolic engineering, molecular biology, and *in silico* tools.

## Introduction

The Global Cancer Observatory database predicted that the anticipated new cancer-related cases would be around 21.6 million globally from 2020 to 2025. There will be a 12.1% rise in new cancer cases, with 11.4 million cases in males and 10.2 million in females by 2025 (Cancer Tomorrow, n.d.). There were an estimated 10 million deaths because of cancer in 2020. According to projected estimates, there will be 47% more cancer cases by 2040, low human development index (HDI) countries will contribute to 95% of cases, and medium HDI countries will contribute to 64% of cases (Sung et al., 2021). By 2025, it is predicted that there will be 11.3 million cancer-related deaths worldwide (Cancer Tomorrow, 2023). Among Indians, the primary cause of death by cancer of the lips and mouth is due to the practice of chewing betel nuts (Gupta et al., 2018). In India, cancer of the oral cavity, digestive system, respiratory system, and genital system indicated a higher projected crude cancer rate for the year 2020 (Mathur et al., 2020). The predicted incidence of new cancer cases in India by the various states and Union territories in 2020 was 1.39 million. It grew to 1.42 million in 2021 and 1.46 million in 2022, according to the National Cancer Registry Programme of the Indian Council of Medical Research (ICMR) report (Mathur et al., 2020; Krishnan et al., 2022; Kumar & Anupama, 2022). India is expected to burden 29.8 million cancer patients by 2025 (Krishnan et al., 2022).

A tumor or neoplasm arises because of uncontrolled cell growth (increase in mass) and the proliferation of an abnormal cell. Benign tumors remain locally confined, and wrecking these abnormal cells cures the disease. Benign tumors do not destroy the local tissue and are non-aggressive. When these abnormal cells become malignant and form secondary tumors called metastases at other body sites, it becomes difficult to eradicate them. Cancer is a malignant tumor in which the cells divide more rapidly. By secreting signal proteins and proteolytic enzymes, they also alter their microenvironment, which comprises connective tissue and inflammatory leukocytes (Feig et al., 2012). Cancer is classified as Carcinoma, sarcoma, leukemia, and lymphoma, depending upon the site of occurrence.

A substantial amount of rare genetic and epigenetic changes in cells derived from single-cell lineage convert them into cancerous outgrowth. These changes arise due to defective DNA damage repair, replication error, chromosomal segregation, and apoptotic machinery, giving the clonal cells the selective advantage of cell proliferation (You and Peter, 2012). Usually, carcinogens, pro-mutagens, and the accumulation of reactive oxygen species are the chemical substances that cause these abnormal mutations or cause tissue damage (Barnes et al., 2018). This tissue damage caused by non-mutagenic carcinogens like alcohol will induce cell proliferation at that site, and due to replication errors, there would be chances of driver mutations to arise, which lead to cancer (Roswall and Weiderpass, 2015).

Overexpression of genes that promote cell proliferation and under-expression of genes that inhibit cell proliferation are the mutations leading to uncontrolled cell growth (Levine & Puzio-Kuter, 2010). Some examples of oncogenes are *K-Ras*, which is involved in receptor tyrosine kinase signaling, and *ß-Catenin*, which is involved in Wnt signaling. Contrarily, tumor suppressor genes include TGF-ß receptor II, which is involved in TGF-ß signaling, Apc, which is involved in Wnt signaling, p53, which is activated in response to abnormal proliferation; and mutated DNA sequences; and Rb, which arrests the cell-cycle transition from G1 to the S phase (Lee & Muller, 2010). Tumor suppressor genes like the *p53* gene positively regulate apoptosis, whereas oncogenes like *Bcl-2* negatively regulate apoptosis (Larsson, 2011). These mutations in cancer cells favor cell proliferation, cell motility, cell invasiveness, and reduced apoptosis.

The clonal evolution of cancer is the process of forming clones of cells with abnormal mutations and developing into true malignancy. The average cancer genome comprises hundreds of mutations. Among all those mutations, there are only 3–15 mutations in tumor suppressors and oncogenes (Bozic et al., 2010; Tomasetti et al., 2015). These are termed driver mutations, as these mutations lead to cancer development, and other mutations are termed passenger mutations (Pao & Girard, 2011). In viruses like the human papillomavirus, the genes *E6* and *E7* encode proteins that prevent the expression of *pRB* and *p53* genes, respectively. Females develop cervical cancer when specific tumor suppressor genes in the cervix are silenced (Moody & Laimins, 2010). Lifestyle changes can reduce cancer risk by eating a healthy diet, exercising, using sunscreen, and avoiding smoking and alcohol. Based on these studies, immunotherapies, gene therapy, targeted therapies against mutated genes in cancer cells, and conventional therapies are some modes of treating cancer.

There is a therapeutic window between the effect of a given concentration of cancer drugs on normal cells and cancer cells. Drugs typically affect cancer cells at a lower concentration, while normal cells require higher concentrations (Dan et al., 2018). By creating cross-links within and between DNA strands, chemotherapy medications like carboplatin and cisplatin cause damage to DNA in cancer cells. Another drug, procarbazine, methylates DNA and prevents replication (Skeel & Khleif, 2011). Clinical usage of cisplatin is associated with several toxicities and side effects; however, the precise mechanism underlying these adverse effects is still unknown (Qi et al., 2019). Chemical drugs like 5-azacytidine, 5-aza-2′ deoxycytidine, 1-β-D-arabinofuranosyl-5-azacytosine, and dihydro-5-azacytidine exert their action by incorporating into the promoter region of tumor suppressor genes and hence reactivating it (Bojang & Ramos, 2014). These nucleoside analogs cannot be constantly administered to patients due to their cytotoxicity and instability, which is a significant drawback.

Histone acetyltransferase inhibitors, like Lys-CoA, work by preventing HAT activity since they are similar molecules to acetyl-CoA and thus inhibit HAT reaction (Lau et al., 2000). Similarly, many histone deacetylase inhibitors like vorinostat and romidepsin are helpful in cell cycle arrest (Fatkins & Zheng, 2008). Since many macromolecule protein complexes and enzymes are involved during epigenetic regulation, targeting these enzymes and protein complexes requires efficient molecular docking strategies to eliminate unintended consequences. The major downside of radiation therapy is the emergence of radioresistance in cancer cells and radiotoxicity in normal cells (Nambiar, Rajamani, and Singh 2011).

Plants have various chemical substances that can be used for medicinal purposes. Many bioactive substances have been utilized as anticancer agents, and most of these are currently the subject of clinical trials. Paclitaxel act by increasing microtubule polymerization, thus inhibiting cell proliferation (Ardalani, Avan, and Ghayour-Mobarhan 2017). However, paclitaxel side effects in treating gynecological cancers include neutropenia and peripheral neuropathy (de Castro et al., 2019). Podophyllotoxin derivatives, especially etoposide and teniposide, act as inhibitors of DNA Topoisomerase II, leading the cell to apoptosis (Gallego-Jara et al., 2020). The hydrophobic property of curcumin continues to be the major obstacle to developing effective medicines for cancer treatment (Ayubi et al., 2019). Parthenolide (PTL) also has low bioavailability due to its poor solubility in water (Karam et al., 2021). Neurotoxicity caused by vinca alkaloids is a significant side effect of using these alkaloids as cancer medication (Arora & Menezes, 2021).

This overview illustrates the biosynthesis of many anticancer compounds, including curcumin, paclitaxel, vinca alkaloids, sulforaphane (SFN), diindolylmethane, PTL, and podophyllotoxin. Several research studies have been highlighted depicting the role of synthetic analogs in increasing the solubility of phytochemical compounds and overcoming the side effects of these phytochemical compounds using various pharmacological approaches. Along with this, we highlighted the structural activity relationships and the mechanism of action of these phytochemical compounds. We also explored metabolic engineering and other related approaches to enhance the levels of anticancer compounds. The last section of this article discusses molecular docking techniques to explore a vast array of secondary metabolites in chemotherapy.

## Anticancer phytochemical compounds and their mechanism of action

### Curcumin and its analogs

Curcumin, a phenolic compound derived from the *Curcuma longa* plant, has been demonstrated to control the expression of various growth factors in cells. Diferuloylmethane or curcumin also regulates the expression of kinases, inflammatory cytokines, transcription factors, cell cycle regulators, and apoptotic proteins (Giordano & Tommonaro, 2019). Curcumin has been used in treating prostate, cervical, uterine, and breast cancer with clinical trials in several trial phases (Giordano & Tommonaro, 2019). In pancreatic cancer, Diferuloylmethane downregulates the expression of *NF-κB, miR-21, c-Myc, Hes-1, Stat-3, COX-2, CD-31, VEGF, Notch* gene and upregulates the expression of p21 and p27 genes thereby suppress tumor growth (Bimonte et al., 2016). Curcumin inhibits the cell cycle in colorectal cancer by upregulating caspases and downregulating cyclin D1 and cyclin D3.

Additionally, it upregulates the expression of apoptotic genes like *Bax* while downregulating inflammation-related genes like *TNF-α*, cytokines, and *NF-κB* (Pricci et al., 2020). Similarly, various cell signal transduction pathways are targeted in breast cancer, lymphoma, leukemia, multiple myeloma, prostate cancer, and brain tumors (Liu and Chen, 2013; Giordano & Tommonaro, 2019). The IC50 value of curcumin using MCF-7 cell line of Human Breast Adenocarcinoma cells at 24, 48, and 72 h was 79.58, 53.18, and 30.78 nM respectively (*p* < 0.05). *Mcl-1* gene product was also suppressed by the action of curcumin (Koohpar et al., 2015). There was a significant difference between inhibitory concentration IC50 value for curcumin and nano curcumin on cancer cell line MDA-MB231 of breast cancer cells, with nano curcumin having IC50 value at lower concentrations than curcumin with *p* < 0.01. Hence, nanotechnology is better for treating human breast cancer (Khosropanah et al., 2016). Moreover, curcumin-encapsulated nano-micelles had better cytotoxicity against cisplatin-resistant human oral cancer due to better cellular localization in these cells (Kumbar et al., 2022). For individuals with benign prostatic hypertrophy, a pilot product evaluation study was conducted to examine the effects of curcumin. Curcumin decreases signs and symptoms and enhances the quality of life. Curcumin has a limited bioavailability, although it is non-etheless used in clinical settings. A combination of curcumin and imatinib reduced nitric oxide levels in a randomized control trial of patients with chronic myeloid leukemia (Table 1). A phase II clinical trial of intestinal adenoma patients revealed no significant clinical response due to poor oral bioavailability (Tomeh et al., 2019).

**TABLE 1:** Different Phytochemical compounds possessing anticancer activity.

Sr. No.	Bioactive compound	Plant source	Clinical trial	Compound activity in cancer cells	Reference
1	Curcumin	Rhizome of *Curcuma longa*	Phase I/II clinical trial	• Regulate NF-κB, JAK/STAT and TGF-ß signaling pathways	Willenbacher et al. (2019)
				• Antioxidant properties	
2	Podophyllotoxin and its derivatives	*Chaerophyllum aurium, Justica heterocarpa, Podophyllum hexandrum*	VP/16 cisplatin; Phase III clinical trial.	• Prevents the conversion of tubulin into microtubules	Zhao et al. (2021), Shen et al. (2022), Motyka et al. (2023)
				• Induces cell apoptosis	
3	Paclitaxel and its analogs	*Taxus brevifolia, Taxus baccata, Taxus cuspidata, Taxus wallichiana, Taxus chinensis, Taxus floridana, Taxus Canadensis, Corylus avellana*	Larotaxel; Phase II clinical trial	• Inhibits the depolymerization of microtubules	Naaz et al. (2019)
			BMS-184476; Phase II clinical trial	• Inhibits *Bcl-2* expression	
			Ortataxel; Phase I clinical trial	• Upregulate *p27* and *p21* gene	
4	Diindoylmenthane	Cauliflower, Cabbage, Mustard, Radish, Broccol, Brussels sprouts	Phase II clinical trial for prostate cancer	• Activation Nrf2 signaling pathway	Wang et al. (2022)
				• Suppresses TGF-ß, Smad2/Smad3 signaling, and Ap-1 transcription factor	
5	Parthenolide and its analogs	Leaves of *Tanacetum parthenium and Chrysanthemum parthenium*	Phase I clinical trial of DMAPT	• Suppresses HDACI-mediated NF-κB activation	(Karam et al., 2021)
				• Downregulates the expression of tubulin carboxypeptidase activity	
				• Increases *ATM* gene expression	
6	Vinca alkaloids	*Catharanthus roseus*	Phase II/III clinical trial: Vincristine in combination with other drugs	• Inhibit microtubule assembly	Škubník et al. (2021), Asati (2022)
7	Resveratol	Roots – *Polygonum cuspidatum* Rhizomes – *Veratrum formosanum* Seeds – *Vitis vinifera*	Phase I/II	• Activates *Nrf2*	Whitlock and Baek 2012; Tian and Liu 2020; Farkhondeh et al. (2020), Carter, D’Orazio, and Pearson 2014
				• Activates *p53*	
				• Inactivate *NF-κB* signaling	
				• Resveratol has activity on enzymes related to oxidative metabolism and there by detoxification of these metabolites	
				• Activates *ATF3* in colorectal cancer cells	
				• Inhibits of *COX-1* activity	
8	Pomiferin	Fruits – *Maclura pomifera*	-	• Inhibits activity of histone deacetylase	Amin et al. (2009), Greenwell and Rahman 2015; Venturelli et al. (2016)
				• Reduces viability of malignant neoplastic glioma cells	
				• Strong antioxidant property	
				• Reduces the expression of S100A6 protein	
9	Thymoquinone	Seeds of *Nigella sativa*	Phase I/II	• Inhibit activity of aconitase enzyme	Amin et al., 2009; Woo et al. (2011), Asaduzzaman Khan et al. (2017)
				• Increases accumulation of ROS.	
				• Upregulate *p21* ^*WAF1*^ and *p27* ^*Kip1*^	
				• Induces apoptotic activity in neoplastic cells	
				• Interfere with DNA structure	
10	Combretastatin A-4 analogs	*Combretum caffrum*	Phase II/III	• Inhibits tubulin polymerization	Shan et al. (2011), Nainwal et al. (2019)
				• Antiangiogenic activity	
				• Inhibits metastasis	
11	Epigallocatechin-3-gallate	Leaves of *Camellia sinensis*	Phase I	• Inhibits *c-Jun* N-terminal Kinase	Min and Taeg Kyu (2014), Amin et al. (2009), H. Zhao et al. (2016)
				• Activate *Rb* tumor suppressor gene	
				• Reduces telomerase activity in small cell lung carcinoma	
				• Inhibition of DNA methyltransferase activity	
				• Downregulate PI3K/AKT signaling	
12	Homoharringtonine	*Cephalotaxus harringtonia*	Phase I/II	• Homoharringtonine cause inhibition of protein synthesis	Amin et al. (2009), Srivastava and Raghuwanshi 2021; Mazumder et al. (2022)
				• Inhibits TMEM16A in lung cancer cells (Transmembrane protein 16A Ca^2+^ dependent chloride channel)	
13	Triptolide and its derivatives	*Tripterygium wilfordii*	Phase I/II	• Induce apoptosis by targetting genes like *5-LOX*, *Bcl-2*, *XIAP* and Estrogen Receptor	Noel et al. (2019)
				• Regulates autophagy process by targetting genes like *HSP70* and *mTOR*.	
				• Inhibits transcription in tumor cells by targeting genes like *RPB1*, *MYC*, *SP1* and *FOS*.	
14	Protopanaxadiol	*Panax ginseng*	-	• Induces caspase dependent apoptosis	C.-Z. Wang et al. (2013), Z. Zhang et al. (2015)
				• Induces G1 cell cycle arrest	
				• Induces ROS production	
				• Activates NF-κB pathway	
				• Induces paraptosis	
15	Bruceantin	*Brucea antidysentrica*	-	• Inhibits activity of (AR-FL) and (AR-V7)	Cuendet and Pezzuto 2004; Moon et al. (2021)
				• Inhibits HSP90 Chaperon function	
				• Inhibits protein synthesis by interfering with activity of peptidyltransferase enzyme	
16	Roscovitie	Cotyledons of *Raphnus sativus*	Phase I/II	• Inhibits CDK/Cyclin E activity	Cicenas et al. (2015)

**FIGURE 1:**
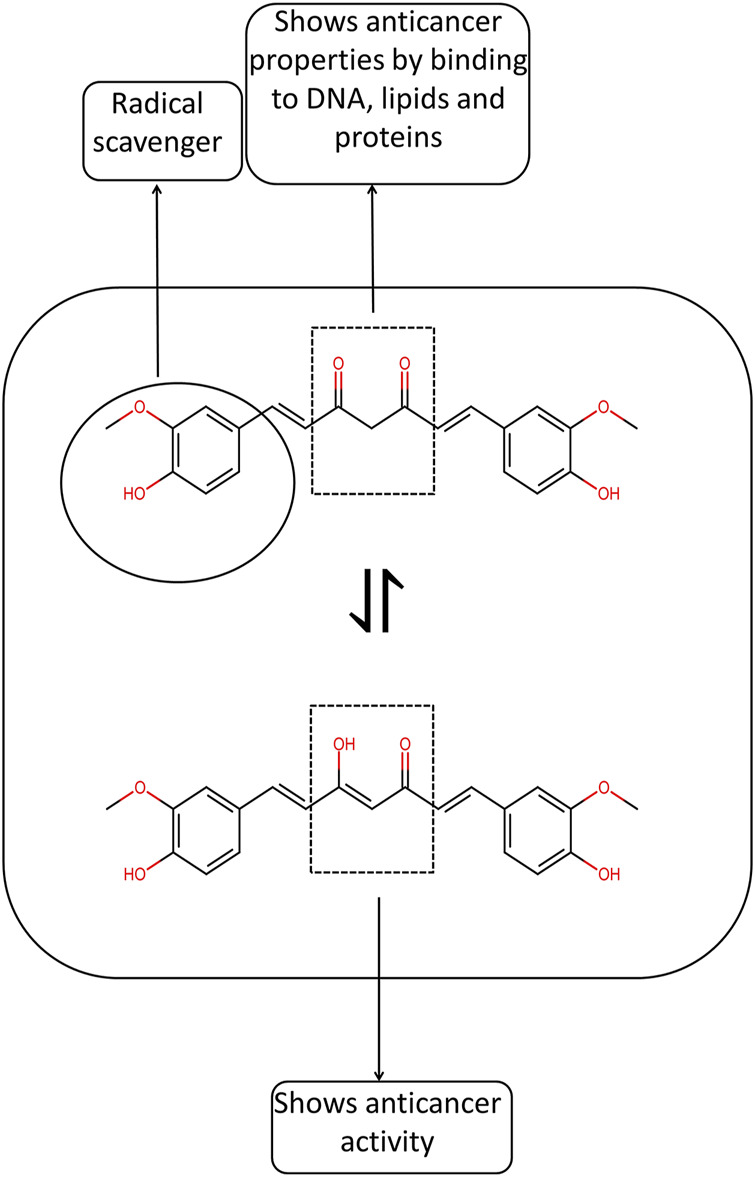
Structural activity relationship of curcumin.

Studies on chemoprevention in murine hepatoma cells showed that the -C=C-C=O moiety in curcumin analogs helps enhance the activation of Phase II detoxifying enzymes. The structural activity relationship (Figure 1) also revealed that, in prostate cancer cells, the conjugated ß-diketone moiety is essential for biological activity (Kaur, Kaur, and Bansal 2021). Adding two ortho-chloro groups in phenyl rings also helps reduce the multiplication of endothelial cells. Introducing the para-methoxy group in the phenyl ring is helpful in the anti-angiogenesis property of curcumin analogs. The anti-angiogenesis property is significantly diminished when the phenyl rings are swapped with pyridyl rings or when the phenyl rings are substituted with ortho chlorine groups (Lin & Lee, 2006). Recently, some curcumin derivatives have been developed where the phenolic–OH groups are entirely or partially substituted, or the diketo chain has been substituted on C4 carbon. Some of these compounds were able to block the expression of NF-κB in triple-negative breast cancer cells more potently than curcumin (Bonaccorsi et al., 2019). Another curcumin derivative, EF24 displayed improved anticancer efficacy compared to curcumin, higher bioavailability, and a slower metabolic rate (Nocito et al., 2021).

### Podophyllotoxin and its analogs

Podophyllotoxin can be extracted from vascular plants belonging to 60 different families, which include genera like *Chaerophyllum aurium* from the Apiaceae family and *Justica heterocarpa* from the Acanthaceae family. (Shah et al., 2021). A critically endangered plant, *Podophyllum hexandrum* of the family Berberidaceae rhizomes, processes several lignans which show anticancer activities (Arora et al., 2010). Various Podophyllotoxin derivatives have been synthesized to address drawbacks like systemic toxicity, drug resistance, and limited bioavailability (H. Fan et al., 2021). Etoposide, teniposide, and etopophos are semi-synthetic anticancer compounds derived from podophyllotoxin. These compounds have fewer side effects than podophyllotoxin (Changxing et al., 2020; Shah et al., 2021; Mishra et al., 2022). Due to systemic toxicity, drug resistance, and low absorption, nearly all podophyllotoxin derivatives were severely constrained in clinical therapy (Zhao et al., 2021). These drugs can efficiently deal with several malignancies, such as Hodgkin’s and non-Hodgkin’s lymphoma, multiform glioblastoma lymphoma, non-lymphocytic leukemia, Wilms tumors, testicular cancer, neuroblastoma and hepatocellular carcinoma (Yu, Che, and Xu 2017; Găman, Egbuna, and Găman 2020). Topoisomerase II activity is inhibited by etoposide, etopophos, and teniposide, which work by creating complexes with DNA and the enzyme. However, the cytotoxicity caused by teniposide is much more than etoposide due to the lesser potency of teniposide and more accumulation of teniposide in cells (B. H. Long 1992). Epipodophyllotoxin is ß confirmation derivative of podophyllotoxin. When 1.2, 3triazole acts as a linker with another pharmacophore moiety, they are categorized as Type II hybrids of epipodophyllotoxin (Xiao et al., 2020). The anticancer property of GL331, NK-611, TOP53, and NPF, as well as other podophyllotoxin compounds such as etopophos and teniposide, are supported by several investigations (Shah et al., 2021). The glucopyranose derivatives of 4-demethylepipodophyllotoxin (DMEP), known as Etoposide (VP-16) and Teniposide (VM-26), are preferred for high-dose chemotherapy treatment of lymphoma, particularly for those patients who experienced recurrence in incurable non-small cell lung cancer (Zhao et al., 2021). The negative consequences of VP16 therapy include genotoxicity and bone marrow suppression. The 3-year overall survival of radiation combined with VP-16/cisplatin was significantly higher than that of paclitaxel/carboplatin in the treatment of non-small cell lung cancer, and the median survival time was 23.3 months, according to the results of a phase III clinical study for the drug combination (Zhao et al., 2021).

Teniposide is more effective than etoposide and causes less damage to hematopoietic stem cells than etoposide (Motyka et al., 2023). 2,6-dimethoxy-4-(6-oxo-(5R,5aR,6,8,8aR, 9-hexahydrofuro [3′,4′:6.7]naphtho[2,3-day] [1,3]dioxol-5-yl)phenyl ((R)-1-amino-4-(methylthio)-1-oxobutan-2-yl)carbamate (DPMA) is a derivative of deoxypodophyllotoxin has more cytotoxic activity compared to etoposide. It also induces *Bax* and inhibits *Bcl-2* expression (Motyka et al., 2023). Multiplication of human cervical adenocarcinoma HeLa cells in *in-vitro* cytotoxicity study was significantly suppressed by the nanoscale drug delivery system Graphene oxide-Polyethylene glycol disulfide prodrug of podophyllotoxin than those of 293T cells, which were taken from normal human kidney. This stemmed from the fact that cancer cells have much greater intracellular glutathione levels than other cells (Y. Liu et al., 2020). Consequently, a redesigned drug delivery mechanism lessens the cytotoxicity of podophyllotoxin derivatives. Hodgkin’s lymphoma patients are treated with etoposide and teniposide using various combinations of other drugs. They can be administered orally or by intravenous route. Myelosuppression, digestive issues, and baldness are toxicities seen during chemotherapy (Găman, Egbuna, and Găman 2020). Aiming the abnormally expressed genes in breast cancer cells, like *p53, cyclin B1, Cdk1, VEGF-A, STAT-3, ERK1/2*, and *AKT-1* podophyllotoxin derivatives like 4ß-amidopodophyllotoxins induce cell cycle arrest. Similarly, by acting on CDK1 and Cyclin B1, podophyllotoxin-norcantharidin hybrids cause cell cycle inhibition and apoptosis (H. Fan et al., 2021).

The IC50 value for accessing the cytotoxicity of podophyllotoxin, picropodophyllotoxin, and deoxypodophyllotoxin (DPT) on colorectal cancer cells by using MTT assay was found to be 649.7, 532.1, and 56.1 nM respectively. In cell lines, namely HT29, DLD1, and Caco2, the IC50 value of DPT was 56.1, 23.4, and 26.9 nM, respectively, at 48 h of drug exposure. This was attributed to the fact that DPT destabilizes microtubules, increases the production of BAX protein, and suppression of production of Bcl-xL protein, leading to programmed cell death (Gamage et al., 2019). Similarly, JNC-1043, a podophyllotoxin derivative, has an IC50 value of 114.5 and 157 nM by using cell viability assay on HCT116 and DLD-1 cell lines of colorectal cancer cell lines at 72 h of drug exposure. Both JNC-1043 and gamma ionizing radiations had effects on increased mitochondrial ROS production (Kwon et al., 2022). For cell lines, namely NCI-H1299 and A549 cells of non-small cell lung cancer cells, the IC50 value of podophyllotoxin acetate was found to be 7.6 and 16.1 nM, respectively, at 72 h of drug exposure. This was due to the inhibition of microtubule polymerization and cell cycle arrest, ultimately leading to apoptosis (Choi et al., 2015).

The structural activity relationship (Supplementary File S1) revealed that both A and E rings are crucial for podophyllotoxin anti-cancer activity. Lactone from the D ring is preferred; the C4 position in the C ring can be modified with bulky groups, and modifications in the B ring are not tolerated (Xiao et al., 2020; X. Zhang et al., 2018; Reddy et al., 2018; Dong et al., 2016). A and D rings can be modified to increase biological activity. E ring shows orthogonal free rotation, which is necessary for the anti-tumor activity of the compound (Nagar et al., 2011).

### Paclitaxel and its analogs

Paclitaxel can be extracted from plants of the Taxaceae family, such as Taxus brevifolia, found in the Western United States; *Taxus baccata*, known as European Yew, *Taxus cuspidata*, known as Japanese Yew; *Taxus wallichiana, Taxus chinensis* *, Taxus floridana, Taxus canadensis* found in the Eastern United States, *Taxus globosa*, and plants of Betulaceae family namely *Corylus avellana* Known as the hazel plant is also a source of paclitaxel (Hodgson, 2012; Swamy et al., 2022). *Taxus wallichiana* is known as Himalayan yew and is located in the temperate Himalayas between 1800 and 3,300 m above sea level, as well as at the height of 1,500 m in the highlands of Meghalaya and Manipur (Juyal et al., 2014). Paclitaxel (generic name) was first synthesized from the bark of *Taxus brevifolia* and is commonly known as Western yew (Juyal et al., 2014). These taxine alkaloids are found in the plant’s leaves, bark, and seeds. Clinically relevant concentrations of paclitaxel cause multipolar spindle fiber formation causing aneuploidy in mitotic tumor cells (Weaver, 2014). In recent studies, it has been confirmed that chromosomal missegregation and cell death were more likely to happen, when spindle multipolarity lasted longer, especially after anaphase began. However, multipolar spindles caused by paclitaxel throughout mitosis can concentrate into bipolar spindles, and thus cells survive. So, paclitaxel efficacy is expected to be increased by therapies that prevent cells from focusing multipolar spindles caused by paclitaxel into bipolar spindles (Scribano et al., 2021).

When administered with carboplatin, paclitaxel has been used successfully to treat ovarian cancer. A lower concentration of paclitaxel causes apoptosis by upregulating the expression of *p21* and *p27* genes. At higher concentrations, paclitaxel causes the stable assembly of microtubules from ß tubulin heterodimers and inhibits their depolymerization. Paclitaxel also increases the activity of the enzyme nicotinamide adenine dinucleotide phosphate oxidase; hence, oxidative stress conditions can be enhanced in ovarian cancer cells. In addition to this, multidrug resistance is alarmingly developing in ovarian cancer patients due to altered gene expression, altered tumor microenvironmen, altered cellular metabolism, altered cellular architecture, and altered cellular processes. All these changes cause drug efflux, reduction of intracellular tubulin concentration, and inhibition of mitosis by activating Raf-1 Kinase (Kampan et al., 2015).

After a protracted development period, paclitaxel finally got clinical approval for treatment against resistant breast cancer in 1994 and ovarian cancer in 1992 (Newman et al., 2008). A phase 3 trial of untreated metastatic triple-negative breast cancer patients received atezolizumab plus abraxane or placebo plus abraxane. With atezolizumab plus abraxane, the patients having metastatic triple-negative breast cancer and programmed death ligand 1 (PD-L1) had a more prolonged progression-free survival (Schmid et al., 2018). PD-L1 is an immune response suppressor, and Atezolizumab inhibits PD-L1 function. Hence this combination is a helpful immune-chemotherapy option for patients with PD-L1+ metastatic or locally progressed TNBC (Kang & Syed, 2020). Hematological toxicity is the major drawback of the treatment of gynecological cancer. Recent research studies revealed that the CYP2C8*3 gene variant was significantly linked with severe (grades 3–4) neutropenia and may be used as a potential indicator of hematological toxicities brought on by paclitaxel/carboplatin therapy. Such a predictive assessment might help with the therapeutic intervention of particular individuals receiving chemotherapy based on paclitaxel (de Castro et al., 2019). In another study, the probiotic formulation SLAB51 effectively increased the expression of opioid and cannabinoid receptors in the spinal cord of CIPN mice. Probiotic use also reduces nerve fiber damage in paws (Cuozzo et al., 2021).

MTT assay was performed to access the IC50 value on HER2 positive cancer cell line BT-474, SKBR3. The IC50 values were 19nM and 4 nM for BT-474 and SKBR3 cell lines of breast cancer. Breast cancer cells with positive HER2 receptors have increased expression of oncomiRs. These factors confer resistance to paclitaxel therapy on HER2-positive breast cancer cells (Haghnavaz et al., 2018). Cytotoxicity on 4T1cell line of human breast cancer cells showed the maximum inhibitory concentration IC50 value of 3.78 mM.

Moreover, paclitaxel encapsulated in cyclodextrin nanoparticles increased the bioavailability of paclitaxel (Varan et al., 2021). The maximal inhibitory concentration of paclitaxel in cervical cells cancer cells lines such as HeLa, ME180, CaSki, SiHa, and C33A was found to be 5.39 ± 0.208, 6.59 ± 0.711, 2.940 ± 0.390nM, 19.303 ± 1.886, and 21.567 ± 2.732 nM respectively.

However, paclitaxel-resistant cervical cancer cell lines had increased the PI3K pathway expression. A combination of paclitaxel with PI3K inhibitors such as BYL-719 and LY294002 showed a synergistic effect (J. J. Liu et al., 2019). The IC50 values of paclitaxel and curcumin mixture on the 4T1 cell line and MDA-MB-231 were found to be 4.05 ± 0.13 and 2.79 ± 0.10 mM, respectively. Moreover, the Paclitaxel curcumin nano drug is more efficient than the paclitaxel and curcumin mixture. These nano drugs had better IC50 value than the curcumin and paclitaxel mixture used in triple-negative breast cancer cell lines (Zuo et al., 2021).

Paclitaxel inhibits the function of Bcl-2, a negative apoptosis regulator, in Kaposi’s sarcoma patients (Tourlaki et al., 2020). In treating various carcinomas, Taxol analog docetaxel has gained immense attention. Docetaxel’s therapeutic application is currently constrained because of its non-specific targeted nature and related side effects (Newman et al., 2008). The plant component 10-deacetylbaccatin-III, an inactive precursor chemical found in the needles of the endangered *Taxus baccata*, is used to synthesize docetaxel artificially (Imran et al., 2020). The low solubility of paclitaxel and docetaxel in water is a significant drawback that results in the low bioavailability of these drugs. Using bio-based nanoparticles such as chitosan helps increase the bioavailability of these drugs (Ashrafizadeh et al., 2020). Many taxol analogs are developed, such as larotaxel dehydrate, paclitaxel polyglumex, cabazitaxel, ortataxel, and taxoperein, are under clinical trials (Newman et al., 2008; Jose, 2020) (Table 1). Phase III clinical trials for treating breast and pancreatic cancer are now being conducted with larotaxel, which features a cyclopropane ring in place of the C-7 hydroxyl group (Newman, Cragg, and Kingston, 2008). It acts on cell lines resistant to paclitaxel and can traverse the blood-brain barrier (Yared and Katherine, 2012). Paclitaxel poliglumex is a nanocarrier for paclitaxel that contains 37% of paclitaxel and is coupled with polyglutamic acid to make it more soluble in water (Newman et al., 2008; Pillai, 2019). A phase III clinical trial using this analog is carried out on ovarian and non-small cell lung cancer patients (Newman et al., 2008). Nanoparticle albumin-bound paclitaxel, DJ-927, Cationic liposomal paclitaxel, polymeric-micelle paclitaxel, DHA-paclitaxel, BMS-184476 are some of the paclitaxel analogs designed to reduce the toxicities mainly neurotoxicity caused by docetaxel and paclitaxel (Newman et al., 2008; Yared and Katherine, 2012).

Structural activity relationship (Supplementary File S2) showed that phenyl moiety at C3′N is necessary for cytotoxicity and antitumor activity. The methoxymethyl group can replace the C7-OH group, and the acetyl group at the C10 position is not necessary for biological activity. Removal of the C2-O-benzyl group reduces biological activity, whereas Ortho and Meta substituted benzyl group increases antitumor activity. Replacement of an eight-member ring with a seven-membered ring can be possible, but an oxetane ring is crucial for compound biological activity (Żwawiak & Zaprutko, 2014)

### Glucosinolates

When the endogenous and exogenous myrosinase hydrolyzes the 4-Methylsulfonylbutyl glucosinolate glucoraphanin, SFN is produced (4-methyl sulfonyl butyl isothiocyanate). The cruciferous vegetables cauliflower, cabbage, mustard, and radish all contain glucosinolates, with broccoli and brussels sprouts having the highest quantities of glucoraphanin (Wang Y. et al., 2016). These glucosinolates are present inactively in plants. At acidic pH, some crucifer plants' epithiospecifier protein (EPS) guides the breakdown of glucoraphanin to produce SFN nitrile. SFN nitrile does not have anti-carcinogenic properties; hence, the downregulation of epithiospecifier protein is necessary to increase sulforaphane production.

Additionally, SFN can be biotransformed reversibly into the erucin metabolite, which affects SFN bioavailability (Bayat Mokhtari et al., 2018). Gut bacteria also possess myrosinase enzyme that converts glucosinolates into isothiocyanate compounds. Gut bacteria in mammals also act on SFN and convert it into an inactive compound (Bayat Mokhtari et al., 2018). Hydrolysis of sinigrin, glucotropaeolin, and gluconasturtiin, glucosinolates leads to the formation of allyl isothiocyanate, benzyl isothiocyanate, and phenyl isothiocyanate, respectively. Upon the action of myrosinase on glucosinolates at basic pH, thiocyanates are formed (Esteve, 2020).

Apoptosis and cell proliferation processes are all regulated by the PI3K-AKT signaling pathway, and in cancer cells, this pathway is predominantly hyperactive (Shi et al., 2019). SFN blocks the function of histone deacetylase, which increases the transcription of genes such as *Bax, BAD,* and *p21* that promote apoptosis. SFE significantly inhibits the PI3K-AKT signaling in lung cancer cells, which results in decreased PTEN expression and reduced phosphorylation of AKT (Yang et al., 2016). SFN can stop CREB from being phosphorylated by MSK2, which inhibits *Bcl-2* and further causes apoptosis in esophageal cancer cells (C. Zhang et al., 2019). There have not been many human clinical trials in China that have looked at the effect of SFN on cancer outcomes, but there have been a lot of Phase 1 human SFN investigations using different SFN sources. These investigations are crucial in developing anticancer drugs (Clarke, Dashwood, and Ho 2008).

The action of myrosinase on glucosinolates also leads to the formation of indoles at neutral pH, which, on coming at acidic pH in the stomach, leads to the formation of diindolylmethane (DIM) (Pawlik, Słomińska-Wojewódzka, and Herman-Antosiewicz 2016; Kołodziejski et al., 2019; Koli et al., 2020; Singla et al., 2021; Williams, 2021). DIM in breast cancer cell lines binds to aryl hydrocarbon receptors. This receptor then binds to the Xenobiotic response element sequence in the cytochrome P450 gene family, thereby rendering them transcriptionally active (Esteve, 2020). These cytochromes modify the molecules present within the cells, activating or deactivating them (Ioannides and Lewis, 2005). DIM also affects the activation of the Nrf2 signaling pathway, thereby leading to the production of proteins involved in detoxification and oxidative stress responses (Thomson et al., 2016). In liver cancer cells, DIM suppresses TGF-ß, Smad2/Smad3 signaling, and Ap-1 transcription factor (Wang S. Q. et al., 2016). DIM also regulates the expression of miRNA, which leads to malignancy. In pancreatic cancer cell line, DIM upregulates the expression of let-7b/c/d/e, miR-200b/c, and miR-146a and downregulates the expression of miR-221 miRNA, thereby controlling tumor progression (Biersack, 2020). The combination of calcium ionophore and DIM resulted in increased apoptosis, increased activation of p-p38 MAPK, and cell proliferation was also significantly inhibited in hepatocellular carcinoma cells (Jiang et al., 2019).

Recently synthetic derivatives of DIM, such as 2.2′-diphenyl-3.3′-diindoylmethane (DPDIM) and another halo, phenyl, and ferrocenyl derivatives with anticancer activity, have been developed. Arsindoline B extracted from Xiamen sea bacterium strain CB101 is a natural DIM derivative that shows anticancer activity. Sterptindole extracted from Streptococcus faecium IB 37 is another natural derivative showing DNA-damaging and genotoxic properties (Pillaiyar et al., 2018). DPDIM has anticancer activity on triple-negative breast cancer cells, lacking estrogen receptors, progesterone receptors, and no HER-2 overexpression (Biersack, 2020). IC50 concentration of C-substituted diindolylmethane derivative, namely DIM10 and DIM14 on triple-negative breast cancer cell line MDA-MB-231, MDA-MB-468, and MDA-MB-435 range from 10 to 20 mM after 72 h of treatment. Nano-structured lipid carriers were used to increase the bioavailability of these drugs (Godugu et al., 2016). Although 3.3′diindoylmethane (DIM) is advocated for its efficiency in the treatment of breast and cancer patients, the majority of the prospective trials focused more on the biological fate of DIM than they did on the effectiveness of DIM in the treatment of breast or prostate cancer (Amare, 2020).

Sulforaphane causes cell cycle arrest and inhibits Akt/mTOR pathway and apoptosis in these cells (Yasunaga et al., 2022). Cytotoxicity of Sulforaphane on Estrogen receptor positive cell lines like T47D and MCF-7 cell lines revealed the half-maximum inhibitory concentration of sulforaphane (IC50 value) was 6.6 and 5 mM, respectively. Both erucin and sulforaphane had similar IC50 values of cytotoxicity on these cell lines. However, IC50 values of cytotoxicity by erucin and sulforaphane showed a significant difference on cell line BT-474, with sulforaphane having cytotoxicity at a less inhibitory concentration than erucin. Co-treatment with 4-hydroxytamoxifen, an estrogen inhibitor, and sulforaphane also sensitizes the 4-hydroxytamoxifen-resistant cell line (Pawlik, Słomińska-Wojewódzka, and Herman-Antosiewicz 2016). For MDA-MB-468 TNBC with overexpressing EGFR, the IC50 value of sulforaphane was found to be 1.8 mM.

### Parthenolide and its analogs

Parthenolide is found in aerial parts, mainly in leaves of *Tanacetum parthenium* and *Chrysanthemum parthenium*. The sesquiterpene lactone family compound 4,5-epoxy-germacra-1 (10),11, (13)-dien-12,6-olide, a secondary metabolite of plants, has three isoprene units and a cyclic ester group (Ghantous et al., 2013). The nucleophilic properties of cyclic ester and epoxide groups enable quick interactions with biological targets. Transcription factor NF-κB is linked to various cellular responses, particularly inflammation, immune regulation, apoptosis, and proliferation. PTL suppresses HDACI-mediated NF-κB activation in acute myeloid leukemia (AML) cells while promoting SAPK/JNK and programmed cell death’s gene activation (Mathema et al., 2012). PTL downregulates the expression of tubulin carboxypeptidase activity; hence, tyrosine ligase can easily tyrosinase tubulins. Healthy neurons and cardiomyocytes have a tubulin detyrosination and retyrosination cycle that regulates microtubule functioning. These highly differentiated cells malfunction when this cycle is dysregulated, and in humans, this can lead to dementia and heart failure (Sanyal et al., 2021). PTL downregulates the expression of DNA methyltransferase enzyme and is less toxic than nucleoside analogs such as 5-azacytidine and decitabine (Ghantous et al., 2013). Additionally, PTL increases *ATM* gene expression, which decreases the activity of the *p21* and histone deacetylase genes. It has been demonstrated that PTL depletes thiols like glutathione (GSH) and blocks related enzymes by alkylation, including GPX1, TXN, TXNRD1/2, and the ligase GCLC1, which accelerates the initial rate-limiting step in the synthesis of GSH. Depletion of glutathione results in the accumulation of ROS and ultimately leads to cell death. PTL also shows anti-proliferative/anti-inflammatory activity. The inhibitory concentration IC50 value for inhibition of *COX-2* and *TNF-α* gene expression by PTL in macrophage cell lines was 0.8 and 0.4 mM, respectively. These genes are involved in inflammatory responses (R. R. A. Freund et al., 2020). Dimethylaminoparthenolide (DMAPT), a PTL analog with improved bioavailability, destroys prostate cancer cells by suppressing NF-κB activity and redox imbalance in the cells (Mendonca et al., 2017) (Table 1). A phase I clinical trial with PTL examined the drug’s pharmacokinetics and toxicity. A phase I clinical trial with DMAPT evaluated cancer patients with acute myeloid leukemia and other blood lymphomas (Sztiller-Sikorska & Czyz, 2020). Dimethylamino-micheliolide fumarate salt (ACT001) is another analog of parthenolide which is under phase I of clinical studies for the treatment of glioma patients. Clinical phase I trials yielded significant findings with acceptable bioavailability and antitumor effectiveness (Sztiller-Sikorska & Czyz, 2020; Lickliter et al., 2021). As *PD-L1* gene transcription is decreased by ACT001, the STAT3 pathway, which is overexpressed and contributes to immunosuppression, can also be reduced (Tong et al., 2020).

The structural activity relationship (Supplementary File S3) showed that the C14 methyl group and the presence of lactam moiety are necessary for the antitumor activity of the compound (R. R. A. Freund et al., 2020; J. Long et al., 2016; Jia et al., 2020). Parthenolide derivative with cinnamyl or substituted cinnamyl moiety is effective in triple-negative breast cancer (Ge et al., 2019). Moreover, parthenolide-5-fluorouracil conjugates are also effective against hepatocellular carcinoma (Ding et al., 2019). A lactone ring is necessary for the compound anticancer activity (R. Freund et al., 2019). Through cytotoxicity assay on cell lines SiHa of human cervical cancer cells and breast cancer cell line MCF-7, the IC50 concentration comes out to be 8.42 ± 0.76 and 9.54 ± 0.82 mM, respectively, with *p* < 0.001. There was over-expression of genes like *p53, Bax, caspase6* and *caspase3* genes. There was also suppression of expression of the *Bcl-2* gene (Al-Fatlawi et al., 2015). The IC50 value of PTL on cancer cell line HCT116 p53+/+ and HCT116 p53−/− of colon carcinoma cells was found to be 17.6 ± 1.8 and 41.6 ± 1.2 mM, respectively. Moreover, the IC50 value for multidrug-sensitive cell lines such as MDA-MB-231-BCRP was found to be 08.5 ± 1.3 mM. This was attributed to the fact that PTL inhibits the NF-κB pathway by binding to IKK and suppressing *HIF-1α* gene expression (Dawood, Ooko, and Efferth 2019).

### Vinca alkaloids and their analogs

Vinca alkaloids from the *Catharanthus roseus* plant include the naturally occurring bis-indole alkaloids vinblastine and vincristine, as well as various semisynthetic equivalents such as vinorelbine, vinflunine, and KAR-2. *Catharanthus roseus* yields the alkaloids vinblastine and vincristine, while *Vinca minor* yields vincamine. Semisynthetic derivatives like vindesine, vinorelbine, and vinflunine are derived from vinblastine and vincristine (Dhyani et al., 2022). Vinblastine, which is primarily used to manage Hodgkin’s disease, and vincristine, which is used in tandem therapy to manage non-lymphoma Hodgkin’s disease, are both now the subject of several human clinical trials (Böll et al., 2011; Škubník et al., 2021). Based on their ability to bind to tubulin, microtubule stabilizing agents can be divided into two groups: those that bind to taxane sites and those that bind to peloruside/laulimalide sites. Vinca domain-binding agents, colchicine domain-binding agents, maytansine site-binding agents, and pironetin site-binding agents are the four categories of microtubule destabilizing agents (Zhang and Kanakkanthara, 2020). Vinca alkaloids inhibit microtubule assembly and cell cycle progression by engaging to α and ß subunits of tubulin (González-Burgos and Gómez-Serranillos 2021). Vinorelbine exhibits neurotoxic effects despite having a stronger affinity for mitotic microtubules than the parent chemical, vincristine. (Milano et al., 2022). Vinblastine and vinorelbine exhibit lower neurotoxicity than vincristine (Verma et al., 2022).

Testicular cancer, Kaposi sarcoma, renal cell carcinoma, lymphoma, and breast and testicular cancer are all treated with vinblastine. One of the main drawbacks of utilizing vinblastine to treat cancer is white blood cell toxicity (Moudi et al., 2013). For non-Hodgkin’s lymphoma, vincristine in combination with medications such as cyclophosphamide, hydroxydaunorubicin, oncovin, and prednisone, as well as for Hodgkin’s lymphoma treatment, mechlorethamine, vincristine, procarbazine, and prednisone are used. The most frequent adverse reactions to vincristine are peripheral neuropathy, decreased bone marrow function, constipation, nervous system toxicity, nausea, and vomiting (Moudi et al., 2013). Vincristine-resistant leukemia and lung cancer cells could be treated with vindesine (Dhyani et al., 2022). Although clinical trials are currently being conducted to determine the effects of neuroprotective medicines against vincristine-induced neuropathy, there is currently no proven treatment for CIPN (Islam et al., 2019; Arora & Menezes, 2021). Vincristine, in combination with other drugs, is under clinical trials for intravascular large B-cell lymphoma and peripheral T-cell lymphoma. The phase II clinical study saw positive outcomes for intravascular large B-cell lymphoma (Table 1). However, in phase II clinical trials, severe toxicities were seen for peripheral T-cell lymphoma (Škubník et al., 2021).

Structural activity relationship (Supplementary File S4) showed that the presence of electron-withdrawing groups is essential for the cytotoxic effect of the compound (Hearn, Shaw, and Myles, 2007; Zheng et al., 2013; Mazumder et al., 2022). The cytotoxicity of vinca alkaloids and other chemically synthesized molecules was evaluated. It was found that the IC50 value of these compounds ranges from 0.89 ± 0.07 to 38.22 ± 5.60 mM in the MCF-7 cell line of breast cancer (Table 1). Among all cell lines tested MCF-7 cell line was most sensitive to chemically synthesized simplified vinca alkaloids. These simplified alkaloids inhibited tubulin polymerization (Zheng et al., 2013).

### Other phytochemical compounds possessing anticancer activity

Several bioactive compounds possessing anticancer activity have been extracted from plant sources (Table 1). A phase I clinical trial on 14 patients of prostate cancer by using resveratrol was done for 2–31 months, depending on the patient’s condition. This clinical study increased prostate-specific antigen doubling time (Paller et al., 2015). Similarly, a phase II clinical trial was performed on 24 patients with colorectal cancer by using resveratrol, but this clinical trial resulted in adverse effects on patients (Popat et al., 2013). Besides these clinical trials, poor pharmacokinetics appears to be the two main constraints of the clinical use of resveratrol (Ren et al., 2021). As histone deacetylase inhibitors, sulforaphane, pomiferin, isothiocyanates, and isoflavones stop the activity of proteins that cause cancer (Greenwell & Rahman, 2015). Thymoquinone was tested in a phase I clinical trial on patients with advanced refractory malignant disease in Arabia, and it was shown to be safe and well-tolerated. However, the dosage did not have anticancer efficacy (Al-Amri & Bamosa, 2009). In phase II randomized clinical trial, thymoquinone decreased the oral premalignant lesions (Clinical and Immunohisochemical Evaluation, 2023). Inflammation caused by hepatitis can also lead to hepatocellular carcinoma. Thymoquinone and bee pollen enhanced liver histology in an *in vitro* rat model of fluvastatin-induced hepatitis (Mohamed et al., 2021). CA-4P (fosbretabulin), a more soluble version of combretastatin-4, exhibited good outcomes in a clinical trial on patients with anaplastic thyroid carcinoma (Mustafa et al., 2022). This water-soluble analog is now in phase II of the clinical trial. Another combretastatin-4 analog, the serine 32 amide AVE8062 (ombrabulin), is undergoing a phase II clinical trial for advanced solid tumors in conjunction with taxanes and platinum salts, as well as a phase II/III clinical trial for patients with advanced soft tissue sarcoma (Zhao et al., 2015).

Based on phase II randomized clinical research, epigallocatechin-3-gallate significantly reduced dermatitis from radiation in breast cancer patients receiving radiotherapy (Zhao et al., 2022). Although several preclinical studies have demonstrated the efficacy of epigallocatechin-3-gallate in the treatment of hepatocellular carcinoma, there is yet to be a clinical trial on actual hepatocellular carcinoma patients (Bimonte et al., 2019). *In vitro* studies showed that the adverse effects of silver nanoparticles on the therapy of Ehrlich ascetic carcinoma could be reduced by exploiting the antioxidant properties of green tea extracts (Magdy et al., 2020). Clinical trials employing homoharringtonine to treat children with acute refractory myeloid leukemia have been reported; however, these trials were ineffective. However, there were notable outcomes when homoharringtonine was used as an induction treatment along with Ara-C and VP-16 (Chen et al., 2019).

Triptolide also has some potential anticancer efficacy, but its main drawbacks are its limited absorption, low solubility, and toxicity. Moreover, much research has yet to be done on how triptolide affects tumor immune infiltration. Minnelide, an analog of triptolide, is currently under phase II clinical trial to treat advanced pancreatic cancer (Noel et al., 2019). Protopanaxadiol is also under Phase I of a clinical study to treat lung cancer and solid tumors (Asati, 2022). Although bruceantin has been shown to have anticancer action using cell cytotoxicity assay, no clinical trials have been conducted as of yet (Asati, 2022). Roscovitine monotherapy in cancer clinical trials has not been very promising, although clinical trials using RSV and sapacitabine in advanced solid tumors are now being conducted (Pandey et al., 2019).

## Bio-active principle and their biosynthetic pathway

### Biosynthesis of curcumin

**FIGURE 2:**
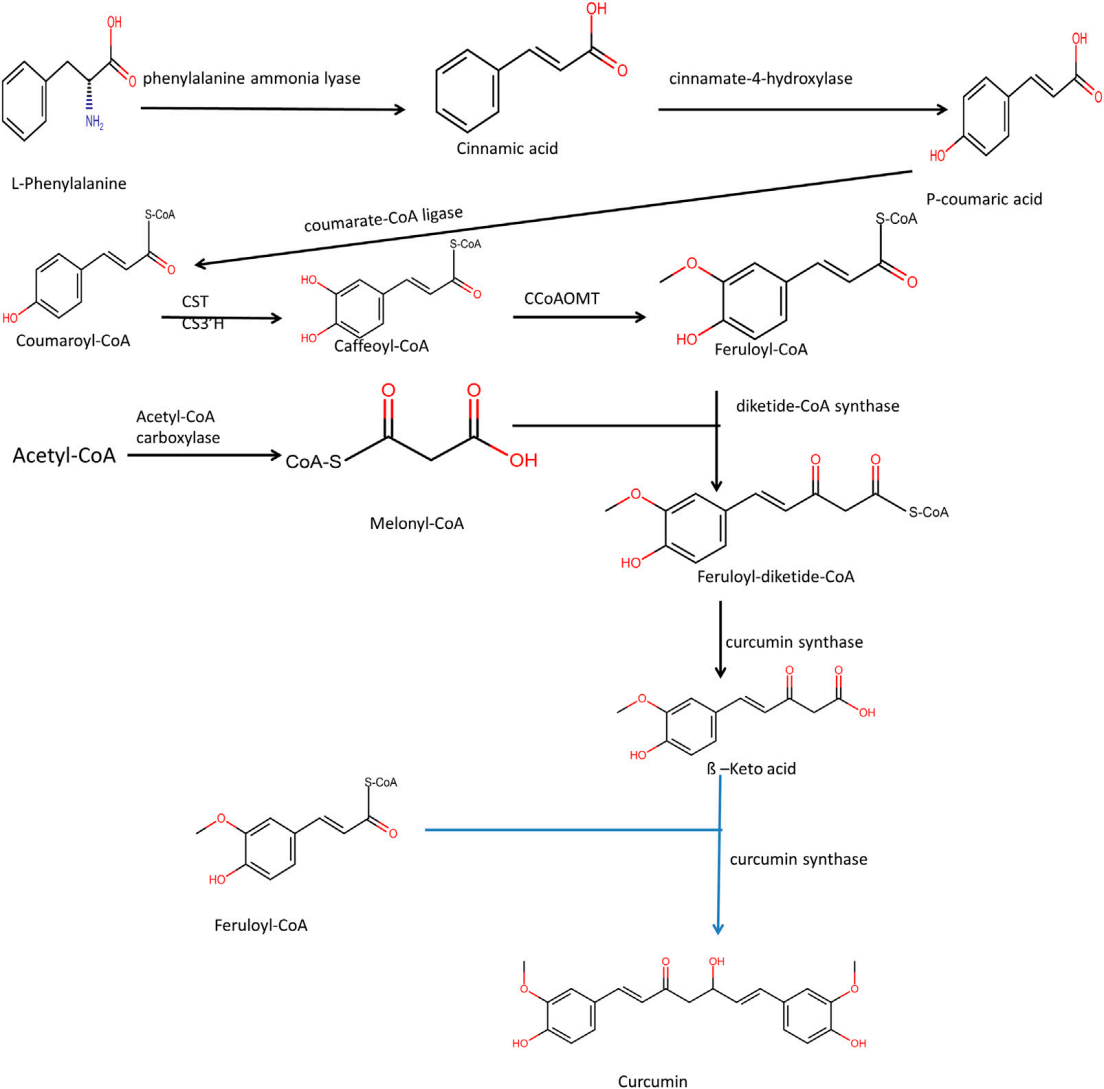
Pathway of Biosynthesis of Curcuminoid. Here CST is p-coumaryl shikimate transferase, CS3′H is p-coumaroyl 5-O-shikimate 3′-hydroxylase and CCoAOMT is caffeoyl-CoA O-methyltransferase.

Curcumin is produced in plants *via* the phenylpropanoid pathway. Release of NH3 from L-phenylalanine takes place by the enzyme phenylalanine ammonia-lyase, and Trans-cinnamic acid gets formed. Cinnamoate-4-hydroxylase participates in the reaction that transforms trans-cinnamic acid into p-coumaric acid. P-coumaric acid is hydrolyzed by 4-coumarate-3-hydroxylase into caffeic acid. Although the transformation of P-coumaric acid to caffeic acid may not be present *in-vivo* in Curcuma longa and P-coumaric acid is directly converted to coumaroyl-CoA by the formation of an activated thioester. Caffeoyl-CoA O-methyltransferase enzyme acts on different substrates such as S-adenosyl-L-methionine and caffeoyl-CoA. The end product of these catalytic reactions is S-adenosyl-L-homocysteine and feruloyl-CoA, respectively. Diketide-CoA is hydrolyzed into the equivalent ß-keto acid by the enzyme diketide-CoA synthase (Figure 2). ß- Ketoacid then gets condensed with feruloyl-CoA by the enzyme curcumin synthase and curcumin forms. Horinouchi and colleagues first reported the enzyme curcumin synthase. The enzyme curcumin synthase couples two molecules of coumaryl-CoA and one malonyl-CoA from the plant *Oryza sativa* to form bisdemethoxycurcumin. Curcumin synthase also catalyzes the reaction of conversion of ß-Keto acid and coumaryl-CoA to form Bisdemethoxycurcumin (Rodrigues et al., 2015). Different metabolic engineering strategies such as deletion of *poxB, curA, *and* adhE* genes, overexpression of *acs* gene, and inactivation of the *fabF* gene have helped improve curcumin’s biosynthesis in a heterologous system (Wu et al., 2020).

### Biosynthesis of epipodophyllotoxin

**FIGURE 3:**
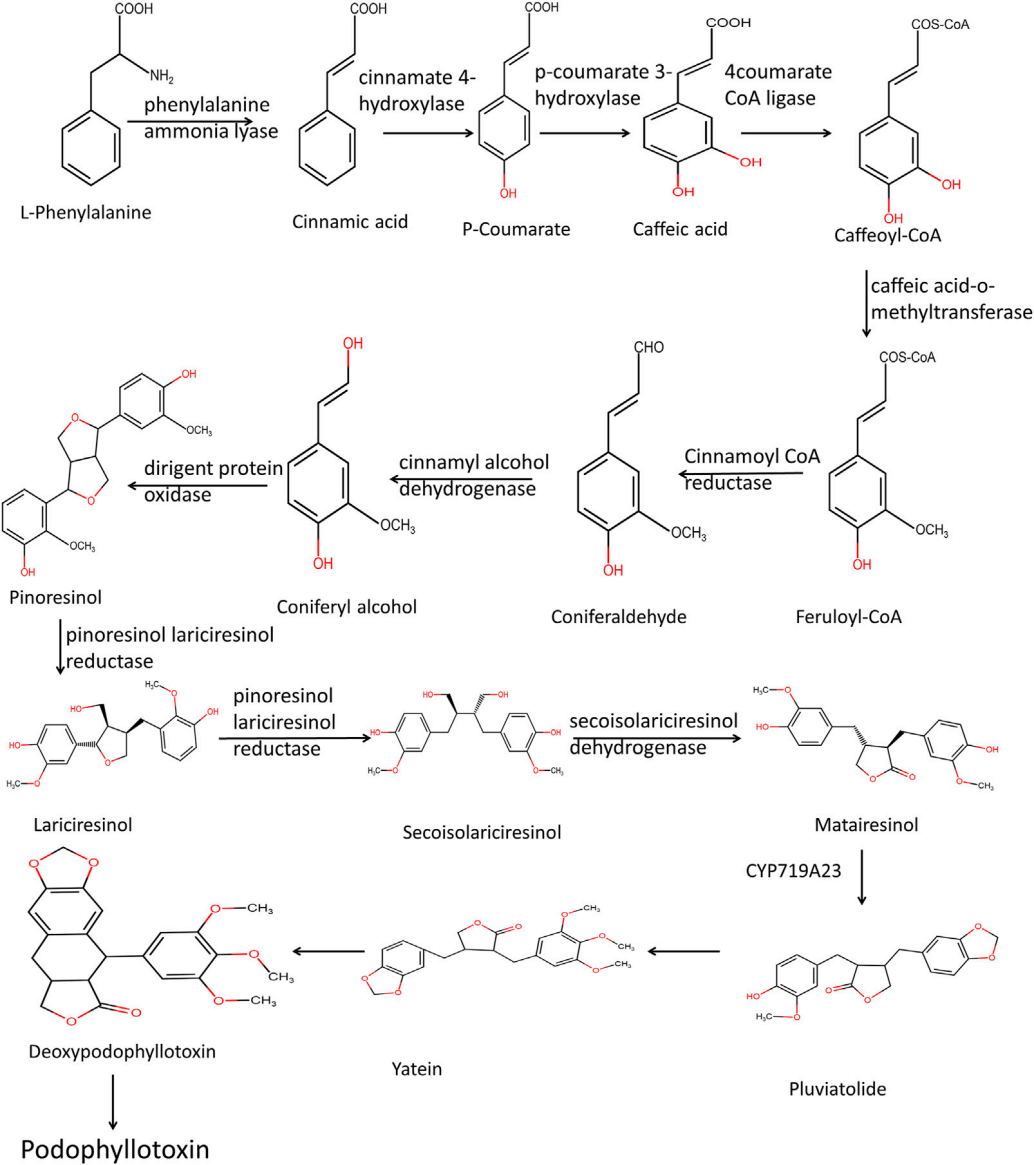
Biosynthesis of podophyllotoxin.

The Biosynthesis of Epipodophyllotoxin takes place through the phenylpropanoid pathway. Both phenylalanine and cinnamic acid are stable precursors for podophyllotoxin biosynthesis (Shah et al., 2021). Phenylalanine deamination takes place with the help of the enzyme phenylalanine ammonia-lyase to form cinnamic acid—a multi-step enzymatic process, including hydroxylation, methyl transfer, reduction, and dehydrogenation from coniferyl alcohol. Two molecules of coniferyl alcohol are combined to form pinoresinol by the enzyme dirigent protein oxidase (Figure 3). From pinoresinol, biosynthesis of diverse lignans takes place through various enzymatic steps. For the synthesis of epipodophyllotoxin, pinoresinol is subsequently reduced and dehydrogenated by enzymes pinoresinol, lariciresinol reductase, and secoisolariciresinol dehydrogenase to form matairesinol. Deoxypodophyllotoxin is hydroxylated to either podophyllotoxin or ß peltatin by the enzymes deoxypodophyllotoxin-7-hydroxylase and deoxypodophyllotoxin-6-hydroxylase, respectively (Yousefzadi et al., 2010). The steps that lead to the formation of deoxypodophyllotoxin are not clear.

### Biosynthesis of paclitaxel

**FIGURE 4:**
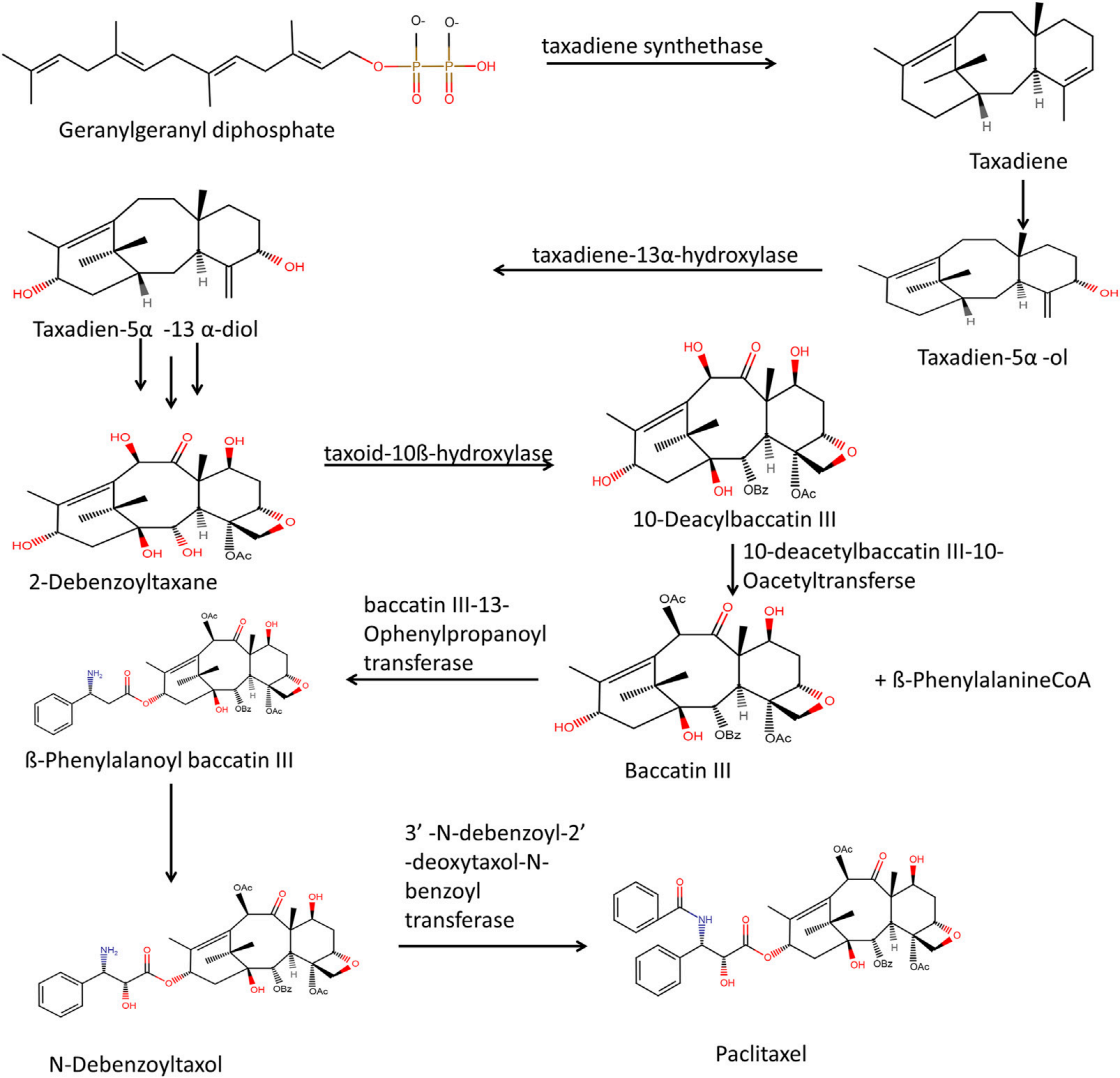
Through sequential steps of mevalonic acid pathway and MEP pathway biosynthesis of Taxadiene takes place. Taxadiene through multistep enzymatic process leads to the formation of paclitaxel.

In plastids, through the methylerythritol phosphate pathway, pyruvate and D-glyceraldehyde 3-phosphate react in multistep enzymatic steps to form isopentenyl pyrophosphate and dimethylallyl pyrophosphate. There is the condensation of three isopentenyl pyrophosphate molecules with one molecule of dimethylallyl pyrophosphate. However, the cytosolic mevalonate pathway also generates precursors such as isopentenyl pyrophosphate and dimethylallyl pyrophosphate, and the formation of paclitaxel can be stopped by inhibitors of both processes (Malik et al., 2011). The cyclization of geranylgeranyl diphosphate (GGPP) to taxa-4 (5), 11 (12) diene4 (taxadiene) is catalyzed by taxadiene synthase, and it marks the beginning of the taxol biosynthesis process. The tricyclic structure formed then goes through several oxygenations and acylations reactions that are region-specific and stereo-specific, carried out by different cytochrome P450 (cP450) oxygenases and acyltransferases. Membrane-bound plant cytochrome P450 called taxadiene-5-Hydroxylase, catalyzes the conversion of taxadiene to taxadiene-5α-ol (Figure 4). Then taxadiene-13α-hydroxylase leads to the conversion of taxadiene-5α-ol to Taxadien-5α -13 α-diol. Through a series of unidentified steps, Taxadien-5α -13 α-diol is converted to 2-Debenzoyltaxane. Hydroxylation reaction converts 2-Debenzoyltaxane to 10- Deacylbaccatin III.

ß-PhenylalanineCoA and Baccatin III in the presence of enzyme baccatin III-13-Ophenylpropanoyl transferase mediate the attachment of ß-PhenylalanineCoA to taxane core. Further acetylation at the C10 position produces Baccatin III, and this reaction is catalyzed by 10-deacetylbaccatin III-10-Oacetyltransferse. By hydroxylation of ß-Phenylalanoyl baccatin III through an unknown c450 enzyme, there is the formation of N-Debenzoyltaxol. The transfer of the benzoyl group to core taxane by enzyme 3′ –N-dibenzoyl-2′ –deoxytaxol-N-benzoyl transferase leads to the formation of the final product paclitaxel (Phillips et al., 2008; Howat et al., 2014; McElroy & Jennewein, 2018; Göbel et al., 2020).

### Biosynthesis of sulforaphane, glucosinolate and glucoraphanin

Glucoraphanin acts as a precursor for the biosynthesis of sulforaphane. In the biosynthetic pathway, there are steps where elongation of the methionine side chain takes place, and other steps include the formation of glucosinolate and side chain modifications of glucosinolate (Supplementary File S5). Through multi-enzymatic steps, methionine gets converted into glucosinolate glucoraphanin. When a plant is injured, the enzyme ß-thioglucosidase, a myrosinase, comes into contact with the glucoraphanin and hydrolyzes it to create the unstable aglucone, which is easily transformed into the isothiocyanate sulforaphane (Supplementary File S6). Core significations in sulforaphane structure also take place through different enzymes in plants. The conversion of methionine to homomethionine takes place in the chloroplast. Flavin monooxygenase converts 4-methylglucosinolate into glucoraphanin (Yagishita et al., 2019; Z. Li et al., 2019; Janczewski, 2022; Nguyen et al., 2020; Neal et al., 2010).

### Biosynthesis of parthenolide

**FIGURE 5:**
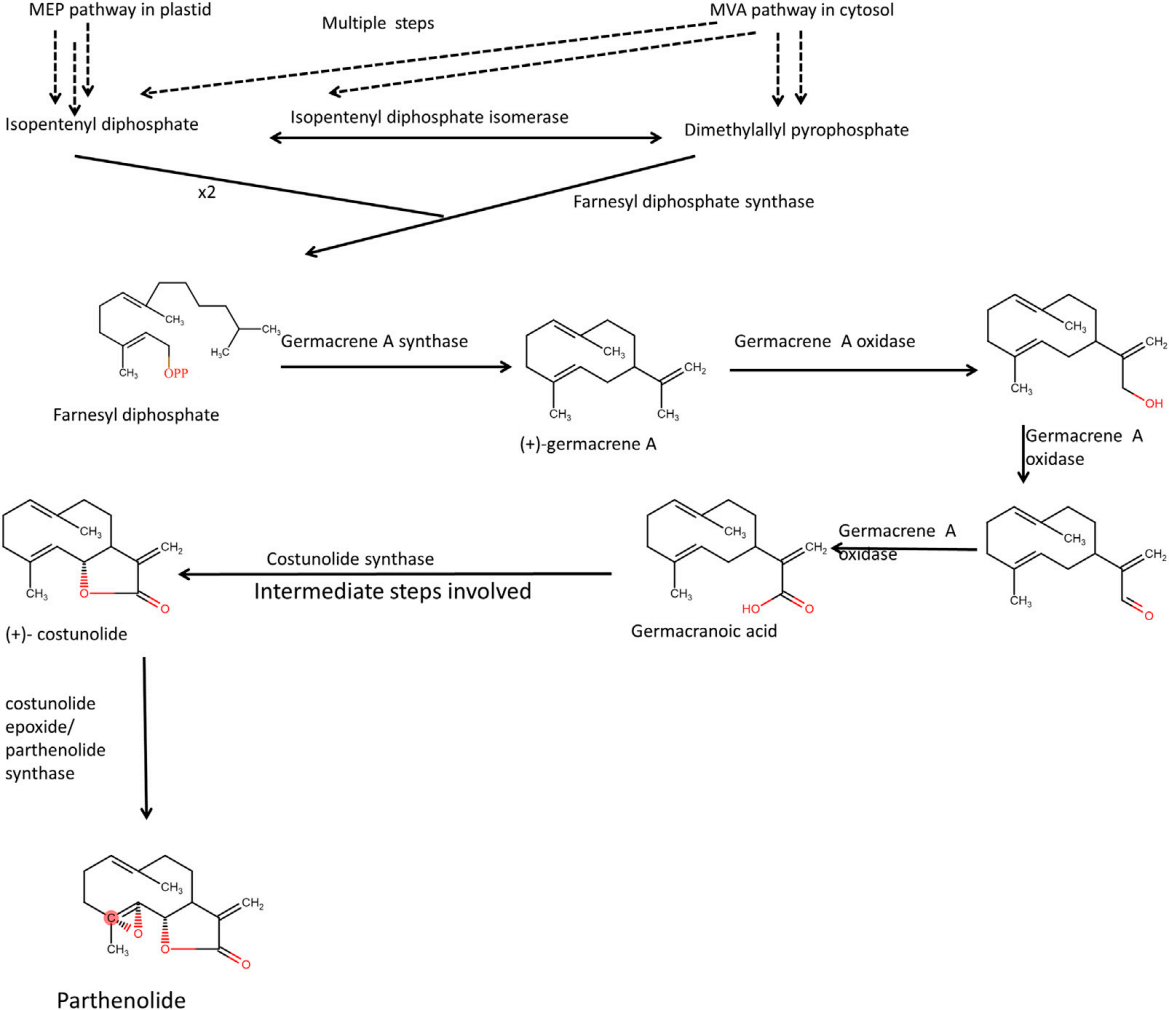
Presumed biosynthetic pathway of parthenolide biosynthesis. Fernesyl diphosphate synthase takes two molecules of isopentenyl pyrophosphate and 1 molecule of dimenthylally pyrophosphate to form farnesyl diphosphate.

The glandular trichomes in feverfew plant flowers contain an excessive amount of parthenolide, and its biosynthesis takes place, presumably by the mevalonate pathway. However, the compounds produced in a heterologous system were conjugated parthenolide (Q. Liu et al., 2014). In the mevalonate pathway, Farnesyl pyrophosphate is formed when two molecules of isopentenyl pyrophosphate and one molecule of dimethylallylpyrophosphate react in the presence of Farnesyl pyrophosphate synthase enzyme. Dimethylallylpyrophosphate can be reversibly changed into isopentenyl pyrophosphate by the enzyme isopentenyl diphosphate isomerase. Germarcene A is formed first, then germacrene A oxidase and Cytochrome P450 work together in a multi-step process to produce germacranoic acid (Figure 5). From costunolide final synthesis of Parthenolide takes place (Majdi et al., 2011; Agabiti et al., 2017). Identification of essential genes involved in the biosynthesis of parthenolide in feverfew plants, such as parthenolide synthase (*TpPT*), germacrene A synthase (*TpGAS*), germacrene A oxidase (*TpGAO*) and costunolide synthase (*TpCOS*) had helped in heterologous expression of parthenolide in Nicotiana benthamiana through metabolic engineering strategies.

### Biosynthesis of vinca alkaloids

The multistep enzymatic process of tryptamine production from chorismate involves the shikimic acid pathway. Anthranilate synthase catalyzes the transfer of the amido group from glutamine in a two-step process to form anthranilate. The formation of pyruvate and glutamate takes place during this reaction. Anthranilate phosphoribosyl anthranilate transferase catalyzes the transformation of anthranilate to 5-phosphoribosyl anthranilate by the transfer of the 5-phosphoribosephosphate group. Through a series of enzymatic steps, there is the formation of indole glycerol phosphate takes place. Both α and ß subunits of the tryptophan synthase enzyme participate in the formation of tryptophan. The tryptophan synthase α subunit catalyzes the formation of the indole ring, and the tryptophan synthase ß subunit adds serine residue to the indole ring, thus forming tryptophan. Tryptophan decarboxylase is an enzyme involved in the decarboxylation reaction that produces tryptamine (Supplementary File S7). Both Mevalonate and Methylerythritol phosphate pathways through multistep enzymatic steps can lead to the formation of geranyl pyrophosphate. This geranyl pyrophosphate is converted to geraniol by the enzyme geraniol synthase. NADPH: cytochrome P450 reductase then acts on geraniol to form 10- hydroxygeraniol. Secologanin is synthesized through a multistep enzymatic process mediated by many cytochrome P450 monooxygenase enzymes, including geraniol 10-hydroxylase, deoxyloganin 7-hydroxylase, and secologanin synthase. When the enzyme strictosidine synthase is present, the reactions between tryptophan and secologanin produce strictosidine during strictosidine biosynthesis (Supplementary File S7). Different terpenoid indole alkaloids can be produced using strictosidine as a precursor. Crystallization of strictosidine produces strictosamide. Further oxidation-recyclization reactions lead to the formation of camptothecin (Sirikantaramas, Yamazaki, and Saito 2013).

Additionally, vinblastine and vindoline are biosynthesized with strictosidine as a precursor. Through hydroxylation and acetyltransferase reactions, the formation of vindoline takes place. Vindoline and catharanthine through acetyltransferase reaction catalyzed by enzyme Deacetylvindoline 4-O-acetyl transferase lead to the formation of vinblastine. The product of vindoline and Catharine coupling is α3′4′anhydrovinblastine, and further, this product is converted into vinblastine (Supplementary File S8). This vinblastine is further converted to vincristine through an unknown enzyme (Sirikantaramas, Yamazaki, and Saito, 2013; Zhu et al., 2015). The enzyme involved in this process is unknown.

## Biotechnological and metabolic engineering used to enhance anticancer compounds production

Heterologous expression of the *Nfstr* gene in *Ophiorrhiza rugosa* (a fast-growing herbaceous plant) leads to enhanced camptothecin production than non-transformed plants. In India, camptothecin is extracted from the tree *Nothapodytes foetida*, and this heterologous expression system can provide an alternative approach to enhancing camptothecin production (Singh et al., 2020). Employing these approaches requires a thorough understanding of the biosynthetic process and the transcriptional profile of the putative genes involved in the pathway. *Agrobacterium*-transformed cells in Nicotiana benthamiana by gene *Mambalgin-1* had significant cytotoxicity against SH-SY5Y cancer cells (nerve cells). Due to this, it may be employed as an anticancer agent (Khezri et al., 2020). Therefore, functional genomics research is essential to understanding how different genes involved in biosynthetic processes are expressed, thereby helping identify those genes whose heterologous expression can enhance the production of beneficial anticancer secondary metabolites.

There is a sharp inclination toward biotechnological approaches because of the complexity and expensive methods for chemical synthesis of Rosmaric acid, which process anticancer, anti-inflammatory, and anti-angiogenesis properties. The use of elicitors in tissue culture, shoot culture, callus culture, cell suspension cultures, optimization methodologies, and bioreactor technologies have all significantly increased rosmarinic acid production (Swamy, Sinniah, and Ghasemzadeh 2018). Moreover, using *Agrobacterium rhizogenes* strain ATCC15834 to transform the hairy root culture of *Dracocephalum kostchyi* and different exposure of iron oxide nanoparticles enhanced the biomass of hairy roots and hence the concentration of rosmarinic acid (Nourozi et al., 2019).

Cell suspension cultures of *Nothapodytes nimmoniana* were supplemented with elicitors such as chitin, chitosan, pullulan, glutathione, and jasmonic acid. This study showed that chitin-treated cell suspension culture had a higher amount of camptothecin with 11.48-fold increases in camptothecin level (Keshavan et al., 2022). Similar methods in *in-vitro* cultures are utilized to manufacture vincristine from *Catharanthus roseus* and podophyllotoxin from *Podophyllum* species in stirred tank bioreactors (Patel et al., 2022). Endophytic fungal species such as *Acremonium, Colletotrichum*, and *Fusarium* with host *Taxus baccata* produced paclitaxel. Using pulverized bark of Taxus baccata as an inducer in a culture medium containing Acremonium, levels of paclitaxel biosynthesis were enhanced.

Moreover, Acremonium has a *BAPT* gene that encodes the protein for paclitaxel biosynthesis (El-Bialy and El-Bastawisy, 2020). By inducing advantageous mutagenesis, those endophytes can be chosen to endure at higher paclitaxel concentrations. This means that the synthesis of paclitaxel employing these endophytic fungi distant from their host plant can be accomplished through microbial fermentation using a variety of elicitors and inducing mutagenesis (El-Bialy and El-Bastawisy, 2020).

It is possible to boost the synthesis of paclitaxel by utilizing elicitors from endophytic fungi (Salehi et al., 2019). *Chaetomium globosum* endophytic fungi cell extract was used as an elicitor in the cell suspension culture of *Corylus avellana*. Additionally, using nanoparticles like coronatine in culture media boosts the expression of genes involved in the generation of taxol, like *BAPT* and *DBTNBT*. Treatment of tissue culture with calix [8]arenes increased the discharge of paclitaxel into the culture medium, and coronatine increased the synthesis of paclitaxel. So using coronatine and calix [8]arenes as elicitors in *in-vitro* cultures of *Taxus media* can enhance the levels of paclitaxel (Escrich et al., 2021). Using A4, A4T, and A8196 strains of Agrobacterium rhizogenes hairy root phenotype of *Curcuma longa* roots was produced with cultures transformed with the A4 strain of *Agrobacterium rhizogenes* showed maximum biomass production. This work used an elicitor like methyl-jasmonate and produced three curcuminoids, namely curcumin, demethoxycurcumin, and bisdemethoxycurcumin, with notable outcomes (Sandhya & Giri, 2022). Moreover, yeast extract and salicylic acid also elicit enhanced curcumin biosynthesis in *in-vitro* cultures (Lan et al., 2019).

Not only plants but endophytic fungi present in plants also process anticancer compounds that can be explored through comparative metabolomics studies (Wei et al., 2020). Cell components from related endophytic fungi may act as elicitors and increase the levels of secondary metabolites obtained from *in-vitro* cultures (Farhadi et al., 2020). Using endophytic fungal cell extracts from *Catharanthus roseus* plants such as Fusarium solani RN1 and *Chaetomium funicola* RN3 and using those extracts as an elicitor in suspension culture augmented the yield of both vincristine and vinblastine plant alkaloid (Linh et al., 2021). Biotic elicitors, such as cell extract from endophytic fungi such as *Alternaria sesami*, were also utilized to boost the levels of alkaloids in the cell suspension culture of *Catharanthus roseus* (Birat et al., 2022).

## Molecular docking studies for anticancer compounds from different plants

The advent of computer-aided drug designing using conventional molecular docking tools and reverse docking tools and the amalgamation of structural molecular biology have carved the path of predicting the binding modes and binding affinity of ligands with different proteins (Fan et al., 2019). Different analogs of curcumin were docked with EGFR receptors. The binding energy of 3,5-Bis (4-hydroxy-3-methoxystyryl)-1H-pyrazole-1-yl-(phenoxy) ethanone and 3,5-bis(4-hydroxy-3-methoxystyryl)-1H-pyrazole-1-yl-(2,4-dichloro phenoxy)ethanone lied in the range of −7.778 and −6.003 kcal/mol (Ahsan et al., 2022). Another study demonstrated the anticancer effects of 20 different *Curcuma longa* compounds using an *in silico* methodology. Out of these 20 compounds, α-curcumene, curcumin, curcumenol, curcumin III, and curcumin II showed binding energy in the range of −5.0 kcal/mol to −7.5 kcal/mol. These compounds were docked with the EGFR, FGFR, and VEGFR-2 receptors (Kusuma et al., 2022). Similarly, several plant-derived phytochemicals, anticancer compounds extracted from the heterologous system, and synthetically and semisynthetically derived anticancer compounds were docked with the putative docking receptors to predict their binding modes (Table 2).

**TABLE 2:** Molecular Docking studies and different aspects considered.

Sr No.	Source	Docking receptor	Ligand	Aspects considered	References
1	*Taxus wallichiana*	EGFR	4-Deacetylbaccatin III; Taxawallin J; Tasumatrol B; Paclitaxel	MTT assay was performed using HepG2, A498, NCI-H226, MDR 2780AD cell line. Tasumatrol B showed significant cytotoxicity compared to other compounds	Qayum et al. (2019)
				Binding energy range: 6.9 kcal/mol to -7.1 kcal/mol	
2	Chemical Synthesis by click chemistry	Topoisomerase	Podophyllotoxin-glycerol triazoles	MTT assay was performed using Du145, A-549, MCF-7 and HeLa cell lines. Molecular docking and cytotoxicity assay showed significant results	Nerella et al. (2021)
3	Compounds were chemically synthesized	Vinca binding domain of tubulin	Compound 5; Compound 6; Compound 7; Compound 8; Vinblastine Anhydrovinblastine	Binding energy lied in range of -12.80 kcal/mol to -7.23 kcal/mol	Quan et al. (2019)
				Compound 7 showed better binding affinity and cytotoxicity	
4	*Tanacetum parthenium*	EGFR	Parthenolide	Parthenolide binds to the active site of EGFR *via* hydrogen bonding and hydrophobic interaction	Li et al. (2020)
5	Members of Cruciferae family	ABCB1 ABCC1	DIM	DIM binds with substrate binding site of ABCB1 and ABCC1 in breast cancer cells	Penta et al. (2021)
6	Fusion conjugate of Curcumin and Dichloroaceta	DYRK2	Curcumin; CMC1; CMC2; CMC3; CMC4; CMC6	Binding energy lied in the range: 21 kJ/mol to -67 kJ/mol	Panda et al. (2022)
				CMC2 conjugate has better binding energy	
				CMC1 and CMC2 reduced growth of human breast cancer cells	
7	*Xylopia vielana*	B-Raf kinase protein	Vieloplain F	ADMET estimation, Bioactivity score, Binding efficiency tested which showed significant results	Hassan et al. (2022)
8	Endophytic fungi *Pestalotiopsis breviseta*	Bcl-2	Paclitaxel extracted from fungus	Binding energy value -13.0061 KJ/mol	Kathiravan et al. (2012)
				Further *in-vitro* studies required	
9	Fungus *Fusarium oxysporum, Penicillium wortmannii*	Point Mutated as well as wild type PIK3CA	Wortmannin extracted from *Fusarium oxysporum*	Resazurin cell growth inhibition assay performed and degree of resistance for sensitive and multidrug-resistant P-glycoprotein/MDR1-overexpressing cancer cell lines was 0.81	Kuete et al. (2015)
10	*Polygonum hydropiper*	Tyrosine Kinase	ß-stiosterol and stigmasterol	MTT assay performed on HeLa, MCF-7, NIH/3T3 cell lines showed promising results	Ayaz et al. (2019)
11	*Cordia sebestena* flowers	E6 protein of HPV16	Hesperetin	MTT cytotoxicity assay on HeLa cell line showed significant results	Prakash et al. (2020)
12	Compounds extracted from different plants	E6 protein of HPV16	Out of colchicine, Curcumin, Daphnoretin, Ellipticine and Epigallocatechin-3-gallate; Daphnoretin has better binding properties than others	Further *in-vitro* studies required	Mamgain et al. (2015)
13	Dr. Dukes phytochemicals and ethnobotanical database	JAK2 protein	Out of different ligands docked ajmalimine showed better binding properties with JAK2 protein	Further *in-vitro* studies required	(Achutha, Pushpa, and Manoj 2021)
14	*Taxus* spp. And also chemical synthesis	CYP3A4	Paclitaxel; Isotaxel; 2’Phosphonoxy methyl etherderivative of Paclitaxel; 2’Phosphonoxy methyl carbonatePaclitaxel	Binding energy lied in the range: 32.63 kJ/mol to-48.28 kJ/mol	Munjal et al. (2022)
				There were both hydrogen bonding and hydrophobic interactions. In case of isotaxel ionic interactions were also there	
15	Compounds were extracted from different plants	MAPK namely	Chrysophanol; Curcumin; Hesperidin; Physcion	All compounds had P38α inhibitory activity based on molecular docking. Considerable cytotoxicity was present at high concentration of drug	Selim et al. (2019)
		P38α			
		ERK2			
		JNK			
		MK3			

Thirteen constituents with good drug-like properties and solid binding affinity were found through molecular docking studies of bioactive Ficus carica constituents, with receptors such as CDK-2, CDK-6, Topoisomerase-I, Topoisomerase-II, Bcl-2, and VEGF-2. Moreover, ß-bourbonene has a better binding affinity with all the potential anticancer drug targets (Gurung et al., 2021). MTT assay on breast cancer cell line revealed that methanolic extract fractions of plant *Clinacanthus nutans* having entadamide C and clinamide D have higher cytotoxic activity than other compounds. Molecular docking studies using receptor molecules, Caspase-3 binding site, and ligand as entadamide C and clinamide D showed favorable binding energy. They can be used as a ligand for Caspase-3 receptors in breast cancer cells (Mutazah et al., 2020). Molecular docking studies on *Jasminum humile* by using two compounds, namely 1-methoxyjasmigenin and 1-methyl-9-aldojasmigenin, showed that these compounds could be used as anticancer drugs targeting Mcl-1 (Mansour et al., 2022). Molecular docking studies on *Raphia taedigera* seed oil possess compounds like 3-Methoxy-2,3-dimethylundec-1-ene, which can be used as a target for VEGFR-2 binding and can be used as an anticancer agent (Umar et al., 2021).

## Conclusion and future perspective

In the entire world, cancer continues to be one of the leading causes of death. Overexpression of oncogenes and down-expression in tumor suppressor genes are the two types of mutations that lead to cancer. In the progression of true malignancy, several mutations in oncogenes and tumor suppressor genes are required. Immunotherapy, gene therapy, radiation therapy, chemotherapy, and surgery are some ways to cure cancer patients. Nowadays, cancer cells have been becoming resistant to chemotherapies and radiation therapies.

Moreover, there are certain levels of toxicity related to these plants derived chemotherapeutic drugs. Hence the development of semisynthetic plant-derived analogs and efficient drug delivery systems using nanotechnology approaches should be considered. By limiting our scope to conventional plants that derive anticancer compounds, other plants should also be checked for their anticancer properties. Endophytic microbes also possess some genes whose expression leads to the synthesis of anticancer compounds. This perspective should also be considered in future research.

Functional genomics, proteomics, and transcriptomics approaches, along with bioinformatics tools such as computer-aided drug designing, would help explore the bioactivity of the newly identified compound and its potential targets. Furthermore, comprehensive clinical trials are advised to evaluate the level of toxicity of plant-derived compounds and to use current pharmacological techniques to lessen their toxicity and adverse effects on normal cells. Molecular docking tools would help ascertain the binding energy of a probable anticancer compound with its receptor and thereby add another compound to the plant-derived anticancer compound catalog.

Metabolic engineering strategies require the introduction of various genes of the biosynthetic pathway into the host and the introduction of enhancer regions so that gene expression can be enhanced, and precursor feeding is also required. Biotechnology and heterologous expression studies of biosynthetic genes would help accelerate anticancer compounds' production. Functional genomics and transcriptomics studies would help to point out the putative functional genes and their expression patterns. Developing secondary metabolites with anticancer capabilities in cell cultures is also increased by applying bio-elicitors and chemical elicitors. The increased generation of secondary metabolites with anticancer characteristics could benefit from bioreactor optimization measures.

## Supplementary material

SUPPLEMENTARY FILE S1 Structural activity relationship of podophyllotoxin

SUPPLEMENTARY FILE S2 Structural activity relationship of paclitaxel

SUPPLEMENTARY FILE S3 Structural activity relationship of parthenolide

SUPPLEMENTARY FILE S4 Structural activity relationship of vinca alkaloids

SUPPLEMENTARY FILE S5 Biosynthetic pathway for the synthesis of aliphatic glucosinolates. Here AT is branched-chain amino acid aminotransferase, HM is homomethionine N-monooxygenase, MM is (methylthio)alkanaldoxime N-monooxygenase, ATL is S-alkyl-thiohydroximate lyase, HBG is N-hydroxythioamide S-beta-glucosyltransferase, ADS is aromatic desulfoglucosinolate sulfotransferase. Here X,Y,Z represent unidentified compounds. From methionine to 2-Oxo-6-methylthiohexanoic acid represent the side chain elongation steps and from 2-oxo-5-methylthiopentanoic acid to glucoiberverin represent the glucosinolate core pathway. From 2-oxo-6-methylthiopentanoic acid to glucoerucin also represent glucosinolate core pathway where synthesis of glucosinolates takes place. 4-Methylthiobutyl glucosinolate (glucoerucin) oxidation leads to formation of glucoraphanin (4-Methylsulfonylbutyl glucosinolate

SUPPLEMENTARY FILE S6 Syntheses of aromatic glucosinolates from Phenylalanine, Tyrosine and Tryptophan. Glucoraphanin (4-Methylsulfonylbutyl glucosinolate) is aliphatic glucosinolate and is derived from Methionine. On hydrolysis of glucoraphanin by myrosinase enzyme sulphoraphene formation takes place. This process takes place when plant is chewed, mechanically damaged, digested in the gut

SUPPLEMENTARY FILE S7 Production of tryptamine uses the shikimic acid route. Secologanin's production utilizes the MEP route Tryptamine and Secologanin both reacts in the presence of strictosidine synthase to form strictosidine. Through multistep biosynthetic process there is formation of camptothecin takes place

SUPPLEMENTARY FILE S8 Biosynthesis of catharanthine, vinblastine and vindoline from strictosidine

## Abbreviation

DIM, 3.3′Diindolylmethane; PTL, Parthenolide; SFN, Sulforaphane; HDI, Human Development Index; CIPN, Chemotherapy-Induced Peripheral Neuropathy; TNBC, Triple Negative Breast Cancer.

## References

[B1] Achutha A. S. Pushpa V. L. Manoj K. B. (2021). Comparative molecular docking studies of phytochemicals as Jak2 inhibitors using autodock and ArgusLab. Mater. Today Proc. 41, 711–716. 10.1016/j.matpr.2020.05.661

[B2] Agabiti S. S. Jin L. Wiemer A. J. (2017). Geranylgeranyl diphosphate synthase inhibition induces apoptosis that is dependent upon GGPP depletion, ERK phosphorylation and caspase activation. Cell Death Dis. 8 (3), e2678. 10.1038/cddis.2017.101 28300835 PMC5386513

[B3] Ahsan M. J. Choudhary K. Ali A. Ali A. Azam F. Almalki A. H. (2022). Synthesis, DFT analyses, antiproliferative activity, and molecular docking studies of curcumin analogues. Plants 11 (21), 2835. 10.3390/plants11212835 36365289 PMC9655326

[B4] Al-Amri A. M. Bamosa A. O. (2009). Phase I safety and clinical activity study of thymoquinone in patients with advanced refractory malignant disease. Shiraz E-Medical J. 10 (3), 107–111.

[B5] Al-Fatlawi A. A. Anees A. Al-Fatlawi A. A. Irshad M. Rahisuddin Ahmad A. (2015). Effect of parthenolide on growth and apoptosis regulatory genes of human cancer cell lines. Pharm. Biol. 53 (1), 104–109. 10.3109/13880209.2014.911919 25289524

[B6] Amare D. E. (2020). <p&gt;Anti-Cancer and other biological effects of a dietary compound 3,3ʹ-diindolylmethane supplementation: A systematic review of human clinical trials</p&gt;. Nutr. Diet. Suppl. 12, 123–137. 10.2147/nds.s261577

[B7] Amin A. Gali-Muhtasib H. Ocker M. Schneider-Stock R. (2009). Overview of major classes of plant-derived anticancer drugs. Int. J. Biomed. Sci. IJBS 5 (1), 1–11. 23675107 PMC3614754

[B8] Ardalani H. Avan A. Ghayour-Mobarhan M. (2017). Podophyllotoxin: A novel potential natural anticancer agent. Avicenna J. Phytomedicine 7 (4), 285–294. PMC5580867 28884079

[B9] Arora R. D. Menezes R. G. (2021). “Vinca alkaloid toxicity,” in StatPearls (StatPearls Publishing). 32491774

[B10] Arora R. Malhotra P. Raman C. Gupta D. Sharma R. Baliga S. (2010). “Indian herbal medicine for cancer therapy and prevention,” in Bioactive foods and extracts, 519–543. 10.1201/b10330-39

[B11] Asaduzzaman Khan M. Tania M. Fu S. Fu J. (2017). Thymoquinone, as an anticancer molecule: From basic research to clinical investigation. Oncotarget 8 (31), 51907–51919. 10.18632/oncotarget.17206 28881699 PMC5584300

[B12] Asati V. (2022). Perspectives of anti-cancer phytoconstituents in pharmacotherapy. Int. J. Med. Pharm. Sci. 12, 1. 10.31782/ijmps.2022.12301

[B13] Ashrafizadeh M. Ahmadi Z. Mohamadi N. Ali Z. Abasi S. Dehghannoudeh G. (2020). Chitosan-based advanced materials for docetaxel and paclitaxel delivery: Recent advances and future directions in cancer theranostics. Int. J. Biol. Macromol. 145, 282–300. 10.1016/j.ijbiomac.2019.12.145 31870872

[B14] Ayaz M. Abdul S. Abdul. W. Junaid M. Ullah F. Khan N. Z. (2019). Cytotoxicity and molecular docking studies on phytosterols isolated from polygonum hydropiper L. Steroids 141, 30–35. 10.1016/j.steroids.2018.11.005 30444979

[B15] Ayubi M. Karimi M. Abdpour S. Rostamizadeh K. Parsa M. Zamani M. (2019). Magnetic nanoparticles decorated with PEGylated curcumin as dual targeted drug delivery: Synthesis, toxicity and biocompatibility study. Mater. Sci. Eng. C 104, 109810. 10.1016/j.msec.2019.109810 31499939

[B16] Barnes J. L. Zubair M. John K. Miriam C. P. Martin F. L. (2018). Carcinogens and DNA damage. Biochem. Soc. Trans. 46 (5), 1213–1224. 10.1042/BST20180519 30287511 PMC6195640

[B17] Bayat Mokhtari R. Baluch N. TinaHomayouni S. Morgatskaya E. Kumar S. Kazemi P. (2018). The role of sulforaphane in cancer chemoprevention and health benefits: A mini-review. J. Cell Commun. Signal. 12 (1), 91–101. 10.1007/s12079-017-0401-y 28735362 PMC5842175

[B18] Biersack B. (2020). 3,3’-Diindolylmethane and its derivatives: Nature-inspired strategies tackling drug resistant tumors by regulation of signal transduction, transcription factors and MicroRNAs. Cancer Drug Resist. (Alhambra, Calif.) 3 (4), 867–878. 10.20517/cdr.2020.53 PMC8992569 35582221

[B19] Bimonte S. Albino V. Piccirillo M. Nasto A. Molino C. Palaia R. (2019). Epigallocatechin-3-gallate in the prevention and treatment of hepatocellular carcinoma: Experimental findings and translational perspectives. Drug Des. Dev. Ther. 13, 611–621. 10.2147/DDDT.S180079 PMC6387605 30858692

[B20] Bimonte S. Barbieri A. Leongito M. Piccirillo M. Giudice A. Pivonello C. (2016). Curcumin AntiCancer studies in pancreatic cancer. Nutrients 8 (7), 433. 10.3390/nu8070433 27438851 PMC4963909

[B21] Birat K. Omar Siddiqi T. Showkat Rasool M. Aslan J. Bansal R. Khan W. (2022). Enhancement of vincristine under *in vitro* culture of Catharanthus roseus supplemented with Alternaria sesami endophytic fungal extract as a biotic elicitor. Int. Microbiol. 25 (2), 275–284. 10.1007/s10123-021-00213-w 34622356

[B22] Bojang P. Ramos K. S. (2014). The promise and failures of epigenetic therapies for cancer treatment. Cancer Treat. Rev. 40 (1), 153–169. 10.1016/j.ctrv.2013.05.009 23831234 PMC3823804

[B23] Böll B. Bredenfeld H. Görgen H. Halbsguth T. Eich H. T. Martin S. (2011). Phase 2 study of PVAG (prednisone, vinblastine, doxorubicin, gemcitabine) in elderly patients with early unfavorable or advanced stage Hodgkin lymphoma. Blood 118 (24), 6292–6298. 10.1182/blood-2011-07-368167 21917759

[B24] Bonaccorsi P. M. Labbozzetta M. Barattucci A. Salerno T. M. G. Poma P. Notarbartolo M. (2019). Synthesis of curcumin derivatives and analysis of their antitumor effects in triple negative breast cancer (TNBC) cell lines. Pharmaceuticals 12 (4), 161. 10.3390/ph12040161 31717764 PMC6958375

[B25] Bozic I. Antal T. Ohtsuki H. Carter H. Kim D. Chen S. (2010). Accumulation of driver and passenger mutations during tumor progression. Proc. Natl. Acad. Sci. 107 (43), 18545–18550. 10.1073/pnas.1010978107 20876136 PMC2972991

[B26] Cancer Tomorrow (2023). Cancer Tomorrow. January 23, 2023, Available at: https://gco.iarc.fr/tomorrow/en/dataviz/tables?types=0&sexes=0&mode=population&group_populations=1&multiple_populations=1&multiple_cancers=0&cancers=39&populations=903_904_905_908_909_935&years=2025_2040&single_unit=500000.

[B27] Carter L. G. D’Orazio J. A. Pearson K. J. (2014). Resveratrol and cancer: Focus on *in vivo* evidence. Endocrine-Related Cancer 21 (3), R209–R225. 10.1530/ERC-13-0171 24500760 PMC4013237

[B28] Changxing L. Galani S. Faiz-ul. H. Rashid Z. Naveed M. Fang D. (2020). Biotechnological approaches to the production of plant-derived promising anticancer agents: An update and overview. Biomed. Pharmacother. 132, 110918. 10.1016/j.biopha.2020.110918 33254434

[B29] Chen X. Tang Y. Chen J. Chen R. Gu L. Xue H. (2019). Homoharringtonine is a safe and effective substitute for anthracyclines in children younger than 2 years old with acute myeloid leukemia. Front. Med. 13 (3), 378–387. 10.1007/s11684-018-0658-4 30635781

[B30] Choi J. Y. Wan G. H. Jeong Hyun C. Kim E. M. Kim J. Jung C-H. (2015). Podophyllotoxin acetate triggers anticancer effects against non-small cell lung cancer cells by promoting cell death via cell cycle arrest, ER stress and autophagy. Int. J. Oncol. 47 (4), 1257–1265. 10.3892/ijo.2015.3123 26314270 PMC4583522

[B31] Cicenas J. Kalyan K. Sorokinas A. Stankunas E. Levy J. Meskinyte I. (2015). Roscovitine in cancer and other diseases. Ann. Transl. Med. 3 (10), 135. 10.3978/j.issn.2305-5839.2015.03.61 26207228 PMC4486920

[B32] Clarke J. D. Dashwood R. H. Ho E. (2008). Multi-targeted prevention of cancer by sulforaphane. Cancer Lett. 269 (2), 291–304. 10.1016/j.canlet.2008.04.018 18504070 PMC2579766

[B33] Clinical and Immunohisochemical Evaluation (2023). Clinical and immunohisochemical evaluation of chemopreventive effect of thymoquinone on oral potentially malignant lesions. - full text view - ClinicalTrials.gov. Retrieved January 31, 2023, Available at: https://clinicaltrials.gov/ct2/show/NCT03208790.

[B34] Cuendet M. Pezzuto J. M. (2004). Antitumor activity of bruceantin: An old drug with new promise. J. Nat. Prod. 67 (2), 269–272. 10.1021/np030304+ 14987068

[B35] Cuozzo M. Castelli V. Avagliano C. Cimini A. d’Angelo M. Cristiano C. (2021). Effects of chronic oral probiotic treatment in paclitaxel-induced neuropathic pain. Biomedicines 9 (4), 346. 10.3390/biomedicines9040346 33808052 PMC8066538

[B36] Dan N. Saini S. Vivek K. K. Khan S. Jaggi M. Yallapu M. M. (2018). Antibody-drug conjugates for cancer therapy: Chemistry to clinical implications. Pharmaceuticals 11 (2), 32. 10.3390/ph11020032 29642542 PMC6027311

[B37] Dawood M. Ooko E. Efferth T. (2019). Collateral sensitivity of parthenolide via NF-?b and HIF-α inhibition and epigenetic changes in drug-resistant cancer cell lines. Front. Pharmacol. 10, 542. 10.3389/fphar.2019.00542 31164821 PMC6536578

[B38] de Castro C. L. da Costa Junior L. C. Lourenço L. V. Seba K. S. da Silva T. S. L. Vianna-Jorge R. (2019). Impact of gene polymorphisms on the systemic toxicity to paclitaxel/carboplatin chemotherapy for treatment of gynecologic cancers. Archives Gynecol. Obstetrics 300 (2), 395–407. 10.1007/s00404-019-05197-7 31123858

[B39] Dhyani P. Quispe C. Sharma E. Amit B. Sati P. Dharam Chand A. (2022). Anticancer potential of alkaloids: A key emphasis to colchicine, vinblastine, vincristine, vindesine, vinorelbine and vincamine. Cancer Cell Int. 22 (1), 206. 10.1186/s12935-022-02624-9 35655306 PMC9161525

[B40] Ding Y. Li S. Ge W. Liu Z. Zhang X. Wang M. (2019). Design and synthesis of parthenolide and 5-fluorouracil conjugates as potential anticancer agents against drug resistant hepatocellular carcinoma. Eur. J. Med. Chem. 183, 111706. 10.1016/J.EJMECH.2019.111706 31553932

[B41] Dong M. Liu F. Zhou H. Zhai S. Yan B. (2016). Novel natural product- and privileged scaffold-based tubulin inhibitors targeting the colchicine binding site. Molecules 21 (10), 1375. 10.3390/molecules21101375 27754459 PMC6273505

[B42] El-Bialy H. A. El-Bastawisy H. S. (2020). Elicitors stimulate paclitaxel production by endophytic fungi isolated from ecologically altered Taxus baccata. J. Radiat. Res. Appl. Sci. 13 (1), 79–87. 10.1080/16878507.2019.1702244

[B43] Escrich A. Almagro L. Moyano E. Cusido R. M. Bonfill M. Hosseini B. (2021). Improved biotechnological production of paclitaxel in Taxus media cell cultures by the combined action of coronatine and calix[8]Arenes. Plant Physiology Biochem. 163, 68–75. 10.1016/j.plaphy.2021.03.047 33819716

[B44] Esteve M. (2020). Mechanisms underlying biological effects of cruciferous glucosinolate-derived isothiocyanates/indoles: A focus on metabolic syndrome. Front. Nutr. 7, 111. 10.3389/fnut.2020.00111 32984393 PMC7492599

[B45] Fan H-Y. Zhu Z-L. Xian H-C. Wang H-F. Chen B-J. Tang Y-J. (2021). Insight into the molecular mechanism of podophyllotoxin derivatives as anticancer drugs. Front. Cell Dev. Biol. 9, 709075. 10.3389/fcell.2021.709075 34447752 PMC8383743

[B46] Fan J. Fu A. Zhang L. (2019). Progress in molecular docking. Quant. Biol. 7 (2), 83–89. 10.1007/s40484-019-0172-y

[B47] Farhadi S. Ahmad M. Safaie N. Sadegh Sabet M. Salehi M. (2020). Fungal cell wall and methyl-β–cyclodextrin synergistically enhance paclitaxel biosynthesis and secretion in Corylus avellana cell suspension culture. Sci. Rep. 10 (1), 5427. 10.1038/s41598-020-62196-4 32214149 PMC7096423

[B48] Farkhondeh T. Silvia Llorens F. Mohammad Pourbagher-Shahri A. Ashrafizadeh M. Samarghandian S. (2020). The therapeutic effect of resveratrol: Focusing on the Nrf2 signaling pathway. Biomed. Pharmacother. 127, 110234. 10.1016/j.biopha.2020.110234 32559855

[B49] Fatkins D. G. Zheng W. (2008). Substituting N(epsilon)-thioacetyl-lysine for N(epsilon)-acetyl-lysine in peptide substrates as a general approach to inhibiting human NAD(+)-dependent protein deacetylases. Int. J. Mol. Sci. 9 (1), 1–11. 10.3390/ijms9010001 19325715 PMC2635597

[B50] Feig C. Gopinathan A. Albrecht N. Chan D. S. Cook N. Tuveson D. A. (2012). The pancreas cancer microenvironment. Clin. Cancer Res. 18 (16), 4266–4276. 10.1158/1078-0432.CCR-11-3114 22896693 PMC3442232

[B51] Freund R. Gobrecht P. Fischer D. Arndt H-D. (2019). Advances in chemistry and bioactivity of parthenolide. Nat. Product. Rep. 37, 541–565. 10.1039/C9NP00049F 31763637

[B52] Freund R. R. A. Gobrecht P. Fischer D. Arndt H.-D. (2020). Advances in chemistry and bioactivity of parthenolide. Nat. Prod. Rep. 37 (4), 541–565. 10.1039/C9NP00049F 31763637

[B53] Gallego-Jara J. Lozano-Terol G. Sola-Martínez R. A. Cánovas-Díaz M. Teresa de Diego P. (2020). A compressive review about Taxol®: History and future challenges. Molecules 25 (24), 5986. 10.3390/molecules25245986 33348838 PMC7767101

[B54] Gamage C. D. B. Park S-Y. Yang Y. Zhou R. Isa T. Woo Kyun B. (2019). Deoxypodophyllotoxin exerts anti-cancer effects on colorectal cancer cells through induction of apoptosis and suppression of tumorigenesis. Int. J. Mol. Sci. 20 (11). 10.3390/ijms20112612 PMC6601030 31141929

[B55] Găman A. M. Egbuna C. Mihnea-Alexandru G. (2020). “Chapter 6 – natural bioactive lead compounds effective against haematological malignancies,” in Phytochemicals as lead compounds for new drug discovery. Editors Chukwuebuka E. Shashank K. Jonathan C. I. Shahira M. E. Saravanan K. (Elsevier), 95–115. 10.1016/B978-0-12-817890-4.00006-8

[B56] Ge W. Xin H. Han F. Liu Z. Wang T. Wang M. (2019). Synthesis and structure-activity relationship studies of parthenolide derivatives as potential anti-triple negative breast cancer agents. Eur. J. Med. Chem. 166, 445–469. 10.1016/j.ejmech.2019.01.058 30739826

[B57] Ghantous A. Sinjab A. Herceg Z. Darwiche N. (2013). Parthenolide: From plant shoots to cancer roots. Drug Discov. Today 18 (17), 894–905. 10.1016/j.drudis.2013.05.005 23688583

[B58] Giordano A. Tommonaro G. (2019). Curcumin and cancer. Nutrients 11 (10), 2376. 10.3390/nu11102376 31590362 PMC6835707

[B59] Göbel A. Rauner M. Hofbauer L. C. TilmanRachner D. (2020). Cholesterol and beyond – the role of the mevalonate pathway in cancer biology. Biochimica Biophysica Acta (BBA) – Rev. Cancer 1873 (2), 188351. 10.1016/j.bbcan.2020.188351 32007596

[B60] Godugu C. Ravi D. Stephen H. S. Singh M. (2016). Novel diindolylmethane derivatives based NLC formulations to improve the oral bioavailability and anticancer effects in triple negative breast cancer. Eur. J. Pharm. Biopharm. 108, 168–179. 10.1016/j.ejpb.2016.08.006 27586082 PMC5725228

[B61] González-Burgos E. Gómez-Serranillos M. P. (2021). “Chapter 4 – Vinca alkaloids as chemotherapeutic agents against breast cancer,” in Discovery and development of anti-breast cancer agents from natural products. Editor Goutam B. (Elsevier), 69–101. Natural Product Drug Discovery. 10.1016/B978-0-12-821277-6.00004-0

[B62] Greenwell M. Rahman P. K. S. M. (2015). Medicinal plants: Their use in anticancer treatment. Int. J. Pharm. Sci. Res. 6 (10), 4103–4112. 10.13040/IJPSR.0975-8232.6(10).4103-12 26594645 PMC4650206

[B63] Gupta S. Gupta R. Sinha D. N. Mehrotra R. (2018). Relationship between type of smokeless tobacco & risk of cancer: A systematic review. Indian J. Med. Res. 148 (1), 56–76. 10.4103/ijmr.IJMR_2023_17 30264755 PMC6172923

[B64] Gurung A. B. Ali M. A. Lee J. Abul Farah M. Al-Anazi K. M. (2021). Molecular docking and dynamics simulation study of bioactive compounds from Ficus carica L. With important anticancer drug targets. PLOS ONE 16 (7), e0254035. 10.1371/journal.pone.0254035 34260631 PMC8279321

[B65] Haghnavaz N. Asghari F. Daniel Elieh A. K. Shanehbandi D. Baradaran B. Kazemi T. (2018). HER2 positivity may confer resistance to therapy with paclitaxel in breast cancer cell lines. Artif. Cells, Nanomedicine, Biotechnol. 46 (3), 518–523. 10.1080/21691401.2017.1326927 28509576

[B66] Hassan S. S. Syed Qamar A. Ali F. Ishaq M. Bano I. Hassan M. (2022). A comprehensive in silico exploration of pharmacological properties, bioactivities, molecular docking, and anticancer potential of vieloplain F from xylopia vielana targeting B-Raf kinase. Molecules 27 (3), 917. 10.3390/molecules27030917 35164181 PMC8839023

[B67] Hearn B. R. Shaw S. J. Myles D. C. (2007). “7.04 – microtubule targeting agents,” in Comprehensive medicinal chemistry IIJohn B taylor and david J triggle (Oxford: Elsevier), 81–110. 10.1016/B0-08-045044-X/00205-4

[B68] Hodgson E. (2012). “Toxicology and human environments,” in Progress in molecular biology and translational science (Elsevier Science). Available at: https://books.google.co.in/books?id=B52yTpIor04C . 10.1016/B978-0-12-415813-9.00001-5 22974735

[B69] Howat S. Park B. Oh I. S. Jin Y-W. Lee E-K. Loake G. J. (2014). Paclitaxel: Biosynthesis, production and future prospects. New Biotechnol. 31 (3), 242–245. 10.1016/j.nbt.2014.02.010 24614567

[B70] Imran M. Saleem S. Chaudhuri A. Ali J. Baboota S. (2020). Docetaxel: An update on its molecular mechanisms, therapeutic trajectory and nanotechnology in the treatment of breast, lung and prostate cancer. J. Drug Deliv. Sci. Technol. 60, 101959. 10.1016/j.jddst.2020.101959

[B71] Ioannides C. Lewis D. F. V. (2005). Cytochromes P450 in the bioactivation of chemicals. Curr. Top. Med. Chem. 4 (16), 1767–1788. 10.2174/1568026043387188 15579107

[B72] Islam B. Lustberg M. Staff N. P. Kolb N. Alberti P. Argyriou A. A. (2019). Vinca alkaloids, thalidomide and eribulin‐induced peripheral neurotoxicity: From pathogenesis to treatment. J. Peripher. Nerv. Syst. 24, S63–S73. 10.1111/jns.12334 31647152

[B73] Janczewski Ł. (2022). Sulforaphane and its bifunctional analogs: Synthesis and biological activity. Molecules 27 (5), 1750. 10.3390/molecules27051750 35268851 PMC8911885

[B74] Jia X. Qi L. Wang S. Zeng B. Du G. Zhang C. (2020). Synthesis, cytotoxicity, and *in vivo* antitumor activity study of parthenolide semicarbazones and thiosemicarbazones. Bioorg. Med. Chem. 28 (13), 115557. 10.1016/j.bmc.2020.115557 32546298

[B75] Jiang Y. Fang Y. Yang Y. Xu X. Wang B. Gu J. (2019). Anti-cancer effects of 3, 3’-diindolylmethane on human hepatocellular carcinoma cells is enhanced by calcium ionophore: The role of cytosolic Ca(2+) and P38 MAPK. Front. Pharmacol. 10, 1167. 10.3389/fphar.2019.01167 31649538 PMC6795059

[B76] Jose W. M. (2020). Taxanes – the backbone of medical oncology. Indian J. Med. Paediatr. Oncol. 41 (02), 221–234. 10.4103/ijmpo.ijmpo_1_20

[B77] Juyal D. Thawani V. Thaledi S. Joshi M. (2014). Ethnomedical properties of Taxus wallichiana zucc. (Himalayan yew). J. Traditional Complementary Med. 4 (3), 159–161. 10.4103/2225-4110.136544 PMC4142453 25161920

[B78] Kampan N. C. Mutsa Tatendae M. McNally O. M. Quinn M. Plebanski M. (2015). Paclitaxel and its evolving role in the management of ovarian cancer. BioMed Res. Int. 2015, 413076. 10.1155/2015/413076 26137480 PMC4475536

[B79] Kang C. Syed Y. Y. (2020). Atezolizumab (in combination with nab-paclitaxel): A review in advanced triple-negative breast cancer. Drugs 80 (6), 601–607. 10.1007/s40265-020-01295-y 32248356

[B80] Karam L. Abou Staiteieh S. Chaaban R. Hayar B. Ismail B. Neipel F. (2021). Anticancer activities of parthenolide in primary effusion lymphoma preclinical models. Mol. Carcinog. 60 (8), 567–581. 10.1002/mc.23324 34101920

[B81] Kathiravan G. Sureban S. M. Sree H. N. Bhuvaneshwari V. Kramony E. (2012). Isolation of anticancer drug TAXOL from pestalotiopsis breviseta with apoptosis and B-cell lymphoma protein docking studies. J. Basic Clin. Pharm. 4 (1), 14–19. 10.4103/0976-0105.109402 24808664 PMC3894731

[B82] Kaur G. Kaur M. Bansal M. (2021). New insights of structural activity relationship of curcumin and correlating their efficacy in anticancer studies with some other similar molecules. Am. J. Cancer Res. 11 (8), 3755–3765. 34522447 PMC8414381

[B83] Keshavan B. Santosh Srinivas N. Muthu Tamizh M. Vairamani M. Raman P. (2022). *In vitro* elicitation of camptothecin by challenging with biotic elicitors in Nothapodytes nimmoniana (J.graham) mabb. South Afr. J. Bot. 144, 325–331. 10.1016/j.sajb.2021.08.039

[B84] Khezri G. Bahram Baghban Kohneh R. Ofoghi H. Seyed Javad D. (2020). Heterologous expression of biologically active mambalgin-1 peptide as a new potential anticancer, using a PVX-based viral vector in Nicotiana benthamiana. Plant Cell, Tissue Organ Cult. (PCTOC) 142 (2), 241–251. 10.1007/s11240-020-01838-x 32836586 PMC7323601

[B85] Khosropanah M. H. Amin D. Nezhadhosseini A. Haghighi A. Hashemi S. Nirouzad F. (2016). Analysis of the antiproliferative effects of curcumin and nanocurcumin in MDA-mb231 as a breast cancer cell line. Iran. J. Pharm. Res. IJPR 15 (1), 231–239. 27610163 PMC4986125

[B86] Koli P. Reena Indurthi H. K. Deepak K. S. (2020). Anticancer activity of 3,3′-diindolylmethane and the molecular mechanism involved in various cancer cell lines. ChemistrySelect 5 (37), 11540–11548. 10.1002/slct.202003137

[B87] Kołodziejski D. Anna P. Hanschen F. S. Pilipczuk T. Tietz F. Kusznierewicz B. (2019). Relationship between conversion rate of glucosinolates to isothiocyanates/indoles and genotoxicity of individual parts of Brassica vegetables. Eur. Food Res. Technol. 245 (2), 383–400. 10.1007/s00217-018-3170-9

[B88] Koohpar K. Z. Entezari M. Movafagh A. Hashemi M. (2015). Anticancer activity of curcumin on human breast adenocarcinoma: Role of mcl-1 gene. Iran. J. Cancer Prev. 8 (3), e2331. 10.17795/ijcp2331 26413251 PMC4581370

[B89] Krishnan S. Chaturvedi M. Das P. Stephen S. Mathur P. (2022). Cancer incidence estimates for 2022 & projection for 2025: Result from national cancer Registry Programme, India. Indian J. Med. Res. 156. 10.4103/ijmr.ijmr_1821_22 PMC10231735 36510887

[B90] Kuete V. Saeed M. E. M. Kadioglu O. Jonas B. Hassan K. Henry Johannes G. (2015). Pharmacogenomic and molecular docking studies on the cytotoxicity of the natural steroid wortmannin against multidrug-resistant tumor cells. Phytomedicine 22 (1), 120–127. 10.1016/j.phymed.2014.11.011 25636880

[B91] Kumar D. P. Anupama A. (2022). Incidence estimate of cancer cases in state/UT of India from 2018 to 2021-v-1, 109–115. 07. 10.33140/IJCRT.07.02.10

[B92] Kumbar V. M. Muddapur U. Abdullatif Bin M. Ali Alshehri S. Mohammed Merae A. Ibrahim Abdullah A. (2022). Curcumin-encapsulated nanomicelles improve cellular uptake and cytotoxicity in cisplatin-resistant human oral cancer cells. J. Funct. Biomaterials 13 (4), 158. 10.3390/jfb13040158 PMC9589971 36278627

[B93] Kusuma S. M. W. Utomo D. H. Susanti R. (2022). Molecular mechanism of inhibition of cell proliferation: An in silico study of the active compounds in <i&gt;Curcuma longa&lt;/i&gt; as an anticancer. J. Trop. Biodivers. Biotechnol. 7 (3), 74905. 10.22146/jtbb.74905

[B94] Kwon J-H. Lee N-G. Ram Kang A. Choi I. Y. Song J. Y. Ahn I-H. (2022). JNC-1043, a novel podophyllotoxin derivative, exerts anticancer drug and radiosensitizer effects in colorectal cancer cells. Molecules 27 (20), 7008. 10.3390/molecules27207008 36296600 PMC9607161

[B95] Lan T. T. P. Nguyen Duc H. Nguyen Ngoc L. Tan Quang H. Trinh Huu T. Le Thi Anh T. (2019). Effect of salicylic acid and yeast extract on curcuminoids biosynthesis gene expression and curcumin accumulation in cells of Curcuma zedoaria. J. Plant Biotechnol. 46 (3), 172–179. 10.5010/JPB.2019.46.3.172

[B96] Larsson L-G. (2011). Oncogene- and tumor suppressor gene-mediated suppression of cellular senescence. Seminars Cancer Biol. 21 (6), 367–376. 10.1016/j.semcancer.2011.10.005 22037160

[B97] Lau O. D. Aliya D. C. Vassilev A. Marzilli L. A. Cotter R. J. Nakatani Y. (2000). P300/CBP-Associated factor histone acetyltransferase processing of a peptide substrate: Kinetic analysis of the catalytic mechanism. J. Biol. Chem. 275 (29), 21953–21959. 10.1074/jbc.M003219200 10777508

[B98] Lee E. Y. H. P. Muller. W. J. (2010). Oncogenes and tumor suppressor genes. Cold Spring Harb. Perspect. Biol. 2 (10), a003236. 10.1101/CSHPERSPECT.A003236 20719876 PMC2944361

[B99] Levine A. J. Puzio-Kuter A. M. (2010). The control of the metabolic switch in cancers by oncogenes and tumor suppressor genes. Science 330 (6009), 1340–1344. 10.1126/SCIENCE.1193494 21127244

[B100] Li X. Huang R. Li M. Zhu Z. Chen Z. Cui L. (2020). Parthenolide inhibits the growth of non-small cell lung cancer by targeting epidermal growth factor receptor. Cancer Cell Int. 20 (1), 561. 10.1186/s12935-020-01658-1 33292235 PMC7686780

[B101] Li Z. Liu Y. Li L. Fang Z. Yang L. Zhuang M. (2019). Transcriptome reveals the gene expression patterns of sulforaphane metabolism in broccoli florets. PLOS ONE 14 (3), e0213902. 10.1371/JOURNAL.PONE.0213902 30908527 PMC6433254

[B102] Lickliter J. D. Jennens R. Lemech C. R. Kichenadasse G. Cai D. Su S. Y.-C. (2021). Phase 1 dose-escalation study of ACT001 in patients with recurrent glioblastoma and other advanced solid tumors. J. Clin. Oncol. 39 (15), 2037. 10.1200/jco.2021.39.15_suppl.2037 33939491

[B103] Lin L. Lee K-H. (2006). Structure-activity relationships of curcumin and its analogs with different biological activities. Stud. Nat. Prod. Chem. 33, 785–812. 10.1016/S1572-5995(06)80040-2

[B104] Linh M. T. Mai N. C. Pham Thi H. Ninh Thi N. Phan Thi Hong T. Ninh Khac B. (2021). Development of a cell suspension culture system for promoting alkaloid and Vinca alkaloid biosynthesis using endophytic fungi isolated from local Catharanthus roseus. Plants 10 (4), 672. 10.3390/plants10040672 33807415 PMC8066771

[B105] Liu D. Chen Z. (2013). The effect of curcumin on breast cancer cells. Jbc 16 (2), 133–137. 10.4048/jbc.2013.16.2.133 PMC3706856 23843843

[B106] Liu J. J. JungHo Y. Hye Won L. Min Wha B. Kim O. Choi Y. J. (2019). Inhibition of phosphatidylinositol 3-kinase (PI3K) signaling synergistically potentiates antitumor efficacy of paclitaxel and overcomes paclitaxel-mediated resistance in cervical cancer. Int. J. Mol. Sci. 20 (14), 3383. 10.3390/ijms20143383 31295843 PMC6679163

[B107] Liu Q. Manzano D. Tanić N. Pesic M. Bankovic J. Pateraki I. (2014). Elucidation and in planta reconstitution of the parthenolide biosynthetic pathway. Metab. Eng. 23, 145–153. 10.1016/j.ymben.2014.03.005 24704560

[B108] Liu Y. Lv X. Xia S. Bingjie H. Huang X. Shi P. (2020). PEGylated Graphene oxide as a nanocarrier of the disulfide prodrug of podophyllotoxin for cancer therapy. J. Nanoparticle Res. 22 (9), 281. 10.1007/s11051-020-05003-5

[B109] Long B. H. (1992). Mechanisms of action of teniposide (VM-26) and comparison with etoposide (VP-16). Seminars Oncol. 19 (2), 3–19. Available at: http://europepmc.org/abstract/MED/1329225 . 1329225

[B110] Long J. Ding Y-H. Wang P-P. Zhang Q. Chen Y. (2016). Total syntheses and structure–activity relationship study of parthenolide analogues. Tetrahedron Lett. 57 (8), 874–877. 10.1016/j.tetlet.2016.01.039

[B111] Magdy A. Sadaka E. Hanafy N. El-Magd M. A. Allahloubi N. El Kemary M. (2020). Green tea ameliorates the side effects of the silver nanoparticles treatment of Ehrlich ascites tumor in mice. Mol. Cell. Toxicol. 16 (3), 271–282. 10.1007/s13273-020-00078-6

[B112] Majdi M. Liu Q. Karimzadeh G. Malboobi M. A. Beekwilder J. Cankar K. (2011). Biosynthesis and localization of parthenolide in glandular trichomes of feverfew (Tanacetum parthenium L. Schulz bip.). Phytochemistry 72 (14), 1739–1750. 10.1016/j.phytochem.2011.04.021 21620424

[B113] Malik S. Cusidó R. M. Hossein Mirjalili M. Moyano E. Palazón J. Bonfill M. (2011). Production of the anticancer drug taxol in Taxus baccata suspension cultures: A review. Process Biochem. 46 (1), 23–34. 10.1016/j.procbio.2010.09.004

[B114] Mamgain S. Sharma P. Pathak R. K. Baunthiyal M. (2015). Computer aided screening of natural compounds targeting the E6 protein of HPV using molecular docking. Bioinformation 11 (5), 236–242. 10.6026/97320630011236 26124567 PMC4464539

[B115] Mansour K. A. Ahmed E. Al-Karmalawy A. A. Lahloub M-F. El-Neketi M. (2022). Cytotoxic effects of extracts obtained from plants of the oleaceae family: Bio-guided isolation and molecular docking of new secoiridoids from Jasminum humile. Pharm. Biol. 60 (1), 1374–1383. 10.1080/13880209.2022.2098346 35961303 PMC9377236

[B116] Mathema V. B. Koh Y-S. Thakuri B. C. Sillanpaa M. (2012). Balkrishna chand thakuri, and mika SillanpääParthenolide, a sesquiterpene lactone, expresses multiple anti-cancer and anti-inflammatory activities. Inflammation 35 (2), 560–565. 10.1007/s10753-011-9346-0 21603970

[B117] Mathur P. Krishnan S. Chaturvedi M. Das P. Kondalli Lakshminarayana S. Stephen S. (2020). Cancer statistics, 2020: Report from national cancer Registry Programme, India. JCO Glob. Oncol. 6, 1063–1075. 10.1200/GO.20.00122 32673076 PMC7392737

[B118] Mazumder K. Asma A. Roy P. Biswas B. Emran Hossain M. Sarkar K. K. (2022). A review on mechanistic insight of plant derived anticancer bioactive phytocompounds and their structure activity relationship. Molecules 27 (9), 3036. 10.3390/molecules27093036 35566385 PMC9102595

[B119] McElroy C. Jennewein S. (2018). “Taxol® biosynthesis and production: From forests to fermenters,” in Biotechnology of natural products. Editors Wilfried S. Bernd Markus L. Matthias W. (Cham: Springer International Publishing), 145–185. 10.1007/978-3-319-67903-7_7

[B120] Mendonca M. S. Turchan W. T. Alpuche M. E. Watson C. N. Estabrook N. C. Chin-Sinex H. (2017). DMAPT inhibits NF-?b activity and increases sensitivity of prostate cancer cells to X-rays *in vitro* and in tumor xenografts *in vivo* . Free Radic. Biol. Med. 112, 318–326. 10.1016/j.freeradbiomed.2017.08.001 28782644 PMC6322835

[B121] Milano W. Milano M. F. Capasso A. (2022). Recent patents on the efficacy and tolerability of vinorelbine in the cancer therapy.

[B122] Min K-J. Taeg Kyu K. (2014). Anticancer effects and molecular mechanisms of epigallocatechin-3-gallate. Integr. Med. Res. 3 (1), 16–24. 10.1016/j.imr.2013.12.001 28664074 PMC5481703

[B123] Mishra A. Mulpuru V. Mishra N. (2022). Exploring the mechanism of action of podophyllotoxin derivatives through molecular docking, molecular dynamics simulation and MM/PBSA studies. J. Biomol. Struct. Dyn., 1–10. 10.1080/07391102.2022.2138549 36307902

[B124] Mohamed A. E. El-Magd M. A. El-Said K. S. El-Sharnouby M. Tousson E. M. Salama A. F. (2021). Potential therapeutic effect of thymoquinone and/or bee pollen on fluvastatin-induced hepatitis in rats. Sci. Rep. 11 (1), 15688. 10.1038/s41598-021-95342-7 34344946 PMC8333355

[B125] Moody C. A. Laimins L. A. (2010). Human papillomavirus oncoproteins: Pathways to transformation. Nat. Rev. Cancer 10 (8), 550–560. 10.1038/nrc2886 20592731

[B126] Moon S. J. Chang Jeong B. Hwa Jin K. Joung Eun L. Kim H-J. Kwon Ghee Y. (2021). Bruceantin targets HSP90 to overcome resistance to hormone therapy in castration-resistant prostate cancer. Theranostics 11 (2), 958–973. 10.7150/thno.51478 33391515 PMC7738850

[B127] Motyka S. Jafernik K. Ekiert H. Sharifi-Rad J. Calina D. Al-Omari B. (2023). Podophyllotoxin and its derivatives: Potential anticancer agents of natural origin in cancer chemotherapy. Biomed. Pharmacother. 158, 114145. 10.1016/j.biopha.2022.114145 36586242

[B128] Moudi M. Go R. Christina Yong Seok Y. Nazre M. (2013). Vinca alkaloids. Int. J. Prev. Med. 4 (11), 1231–1235. 24404355 PMC3883245

[B129] Munjal N. S. Shukla R. Singh T. R. (2022). Physicochemical characterization of paclitaxel prodrugs with cytochrome 3A4 to correlate solubility and bioavailability implementing molecular docking and simulation studies. J. Biomol. Struct. Dyn. 40 (13), 5983–5995. 10.1080/07391102.2021.1875881 33491578

[B130] Mustafa M. A Mostafa Y. E Abd Elbaky A. Mohamed M. Abdelhamid D. Mn Abdelhafez E. (2022). Combretastatin A-4 analogs: Past, present, and future directions. Octahedron Drug Res. 1 (1), 0–64. 10.21608/odr.2022.156180.1008

[B131] Mutazah R. Abd Hamid H. Aizi Nor M. R. Mohd Fadhlizil Fasihi Mohd A. Mashitah M. Y. (2020). *In vitro* cytotoxicity of Clinacanthus nutans fractions on breast cancer cells and molecular docking study of sulphur containing compounds against caspase-3. Food Chem. Toxicol. 135, 110869. 10.1016/j.fct.2019.110869 31626839

[B132] Naaz F. Haider M. R. Shafi S. Yar M. S. (2019). Anti-tubulin agents of natural origin: Targeting taxol, vinca, and colchicine binding domains. Eur. J. Med. Chem. 171, 310–331. 10.1016/j.ejmech.2019.03.025 30953881

[B133] Nagar N. Rakesh K. J. Saharan R. Verma S. Sharma D. Bansal K. (2011). Podophyllotoxin and their glycosidic derivatives. Pharmacophore 2 (2), 87–97. Available at: http://www.pharmacophorejournal.com/ .

[B134] Nainwal L. M. Alam M. M. Shaquiquzzaman M. Marella A. Ahmed K. (2019). Combretastatin-based compounds with therapeutic characteristics: A patent review. Expert Opin. Ther. Pat. 29 (9), 703–731. 10.1080/13543776.2019.1651841 31369715

[B135] Nambiar D. Rajamani P. Singh R. P. (2011). Effects of phytochemicals on ionization radiation-mediated carcinogenesis and cancer therapy. Mutat. Research/Reviews Mutat. Res. 728 (3), 139–157. 10.1016/j.mrrev.2011.07.005 22030216

[B136] Neal C. S. Fredericks D. P. Griffiths C. A. Alan D. N. (2010). The characterisation of AOP2: A gene associated with the biosynthesis of aliphatic alkenyl glucosinolates in arabidopsis thaliana. BMC Plant Biol. 10 (1), 170. 10.1186/1471-2229-10-170 20699011 PMC3095303

[B137] Nerella S. Kankala S. Gavaji B. (2021). Synthesis of podophyllotoxin-glycosyl triazoles via click protocol mediated by silver (I)-N-heterocyclic carbenes and their anticancer evaluation as topoisomerase-II inhibitors. Nat. Prod. Res. 35 (1), 9–16. 10.1080/14786419.2019.1610958 31210060

[B138] Newman D. J. Cragg G. M. Kingston D. G. I. (2008). “Chapter 8—natural products as pharmaceuticals and sources for lead structures,” in The practice of medicinal chemistry Editor Camille Georges W. Third Edition (New York: Academic Press), 159–186. 10.1016/B978-0-12-374194-3.00008-1

[B139] Nguyen V. P. T. Stewart J. Lopez M. Ioannou I. Allais F. (2020). Glucosinolates: Natural occurrence, biosynthesis, accessibility, isolation, structures, and biological activities. Molecules 25 (19), 4537. 10.3390/molecules25194537 33022970 PMC7582585

[B140] Nocito M. C. De Luca A. Prestia F. Avena P. La Padula D. Zavaglia L. (2021). Antitumoral activities of curcumin and recent advances to ImProve its oral bioavailability. Biomedicines 9 (10), 1476. 10.3390/biomedicines9101476 34680593 PMC8533288

[B141] Noel P. Hoff D. D. V. Ashok K. S. Velagapudi M. Borazanci E. Han H. (2019). Triptolide and its derivatives as cancer therapies. Trends Pharmacol. Sci. 40 (5), 327–341. 10.1016/j.tips.2019.03.002 30975442

[B142] Nourozi E. Hosseini B. Maleki R. Babak Abdollahi M. (2019). Iron oxide nanoparticles: A novel elicitor to enhance anticancer flavonoid production and gene expression in Dracocephalum kotschyi hairy-root cultures. J. Sci. Food Agric. 99 (14), 6418–6430. 10.1002/jsfa.9921 31294466

[B143] Paller C. J. Rudek M. A. Zhou X. C. Wagner W. D. Hudson T. S. Anders N. (2015). A phase I study of muscadine grape skin extract in men with biochemically recurrent prostate cancer: Safety, tolerability, and dose determination. Prostate 75 (14), 1518–1525. 10.1002/pros.23024 26012728 PMC4537354

[B144] Panda S. S. Tran Q. L. Rajpurohit P. Pillai G. G. Thomas S. J. Bridges A. E. (2022). Design, synthesis, and molecular docking studies of curcumin hybrid conjugates as potential therapeutics for breast cancer. Pharmaceuticals 15 (4), 451. 10.3390/ph15040451 35455448 PMC9028889

[B145] Pandey V. Ranjan N. Narne P. Babu P. P. (2019). Roscovitine effectively enhances antitumor activity of temozolomide *in vitro* and *in vivo* mediated by increased autophagy and Caspase-3 dependent apoptosis. Sci. Rep. 9 (1), 5012. 10.1038/s41598-019-41380-1 30899038 PMC6428853

[B146] Pao W. Girard N. (2011). New driver mutations in non-small-cell lung cancer. Lancet Oncol. 12 (2), 175–180. 10.1016/S1470-2045(10)70087-5 21277552

[B147] Patel P. Patel V. Modi A. Kumar S. Shukla Y. M. (2022). Phyto-factories of anti-cancer compounds: A tissue culture perspective. Beni-Suef Univ. J. Basic Appl. Sci. 11 (1), 43. 10.1186/s43088-022-00203-5

[B148] Pawlik A. Słomińska-Wojewódzka M. Herman-Antosiewicz A. (2016). Sensitization of estrogen receptor-positive breast cancer cell lines to 4-hydroxytamoxifen by isothiocyanates present in cruciferous plants. Eur. J. Nutr. 55 (3), 1165–1180. 10.1007/s00394-015-0930-1 26014809 PMC4819954

[B149] Penta D. Mondal P. Natesh J. Meeran S. M. (2021). Dietary bioactive diindolylmethane enhances the therapeutic efficacy of centchroman in breast cancer cells by regulating ABCB1/P-gp efflux transporter. J. Nutr. Biochem. 94, 108749. 10.1016/j.jnutbio.2021.108749 33910062

[B150] Phillips M. A. León P. Albert B. Rodríguez-Concepción M. (2008). The plastidial MEP pathway: Unified nomenclature and resources. Trends Plant Sci. 13 (12), 619–623. 10.1016/j.tplants.2008.09.003 18948055

[B151] Pillai G. (2019). “Chapter 9 – nanotechnology toward treating cancer: A comprehensive review,” in Applications of targeted nano drugs and delivery systems. Editors Mohapatra S. S. Ranjan S. Dasgupta N. Mishra R. K Thomas S. (Elsevier), 221–256. Micro and Nano Technologies. 10.1016/B978-0-12-814029-1.00009-0

[B152] Pillaiyar T. Gorska E. Gregor S. Müller C. E. (2018). General synthesis of unsymmetrical 3,3′-(Aza)Diindolylmethane derivatives. J. Org. Chem. 83 (17), 9902–9913. 10.1021/acs.joc.8b01349 30025207

[B153] Popat R. Plesner T. Davies F. Cook G. Cook M. Elliott P. (2013). A phase 2 study of SRT501 (resveratrol) with bortezomib for patients with relapsed and or refractory multiple myeloma. Br. J. Haematol. 160 (5), 714–717. 10.1111/bjh.12154 23205612

[B154] Prakash S. Elavarasan N. Subashini K. Kanaga S. Dhandapani R. Sivanandam M. (2020). Isolation of hesperetin – a flavonoid from Cordia sebestena flower extract through antioxidant assay guided method and its antibacterial, anticancer effect on cervical cancer via *in vitro* and *in silico* molecular docking studies. J. Mol. Struct. 1207, 127751. 10.1016/j.molstruc.2020.127751

[B155] Pricci M. Girardi B. Giorgio F. Losurdo G. Ierardi E. Alfredo Di L. (2020). Curcumin and colorectal cancer: From basic to clinical evidences. Int. J. Mol. Sci. 21 (7), 2364. 10.3390/ijms21072364 32235371 PMC7178200

[B156] Qayum M. Nisar M. Rauf A. Khan I. Kaleem W. A. Raza M. (2019). *In-vitro* and *in-silico* anticancer potential of taxoids from Taxus wallichiana Zucc. Biol. Futura 70 (4), 295–300. 10.1556/019.70.2019.33 34554543

[B157] Qi L. Luo Q. Zhang Y. Jia F. Zhao Y. Wang F. (2019). Advances in toxicological research of the anticancer drug cisplatin. Chem. Res. Toxicol. 32 (8), 1469–1486. 10.1021/acs.chemrestox.9b00204 31353895

[B158] Quan P. M. Binh V. N. Ngan V. T. Trung N. T. Anh N. Q. (2019). Molecular docking studies of Vinca alkaloid derivatives on Tubulin. Vietnam J. Chem. 57 (6), 702–706. 10.1002/vjch.201900087

[B159] Reddy V. G. Reddy Bonam S. Reddy T. S. Akunuri R. Naidu V. G. M. Nayak V. L. (2018). 4β-Amidotriazole linked podophyllotoxin congeners: DNA topoisomerase-iiα inhibition and potential anticancer agents for prostate cancer. Eur. J. Med. Chem. 144, 595–611. 10.1016/j.ejmech.2017.12.050 29289884

[B160] Ren B. Kwah M. X.-Y. Liu C. Ma Z. Shanmugam M. K. Ding L. (2021). Resveratrol for cancer therapy: Challenges and future perspectives. Cancer Lett. 515, 63–72. 10.1016/j.canlet.2021.05.001 34052324

[B161] Rodrigues J. L. Prather K. L. J. Kluskens L. D. Rodrigues L. R. (2015). Heterologous production of curcuminoids. Microbiol. Mol. Biol. Rev. 79 (1), 39–60. 10.1128/MMBR.00031-14 25631288 PMC4402967

[B162] Roswall N. Weiderpass E. (2015). Alcohol as a risk factor for cancer: Existing evidence in a global perspective. J. Prev. Med. Public Health = Yebang Uihakhoe Chi 48 (1), 1–9. 10.3961/jpmph.14.052 25652705 PMC4322512

[B163] Salehi M. Ahmad M. Safaie N. Farhadi S. (2019). Elicitors derived from endophytic fungi Chaetomium globosum and paraconiothyrium brasiliense enhance paclitaxel production in Corylus avellana cell suspension culture. Plant Cell, Tissue Organ Cult. (PCTOC) 136 (1), 161–171. 10.1007/s11240-018-1503-9

[B164] Sandhya S. Giri A. (2022). “Development of efficient Agrobacterium rhizogenes-mediated hairy root system in Curcuma longa L. And elicitation driven enhanced production of pharmaceutically important curcuminoids,” *in Vitro* cellular & developmental biology – plant. 10.1007/s11627-022-10298-1

[B165] Sanyal C. Pietsch N. Rios S. R. Peris L. Lucie C. Marie-Jo M. (2021). “The detyrosination/Re-tyrosination cycle of tubulin and its role and dysfunction in neurons and cardiomyocytes. Seminars Cell & Dev. Biol. 10.1016/j.semcdb.2021.12.006 34924330

[B166] Schmid P. Adams S. Rugo H. S. Schneeweiss A. Barrios C. H. Iwata H. (2018). Atezolizumab and nab-paclitaxel in advanced triple-negative breast cancer. N. Engl. J. Med. 379 (22), 2108–2121. 10.1056/NEJMoa1809615 30345906

[B167] Scribano C. M. Wan J. Esbona K. Tucker J. B. Lasek A. Zhou A. S. (2021). Chromosomal instability sensitizes patient breast tumors to multipolar divisions induced by paclitaxel. Sci. Transl. Med. 13 (610), eabd4811. 10.1126/scitranslmed.abd4811 34516829 PMC8612166

[B168] Selim N. M. Elgazar A. A. Abdel-Hamid N. M. El-Magd M. R. A. Yasri A. Hefnawy H. M. E. (2019). Chrysophanol, physcion, hesperidin and curcumin modulate the gene expression of pro-inflammatory mediators induced by LPS in HepG2: *In silico* and molecular studies. Antioxidants (Basel, Switz. 8 (9), 371. 10.3390/antiox8090371 PMC6770650 31484451

[B169] Shah Z. Umar Farooq G. Jamshed I. Mushtaq A. Mukhtar H. Muhammad Z-U-H. (2021). Podophyllotoxin: History, recent advances and future prospects. Biomolecules 11 (4), 603. 10.3390/biom11040603 33921719 PMC8073934

[B170] Shan Y. Zhang J. Liu Z. Wang M. Dong Y. (2011). Developments of combretastatin A-4 derivatives as anticancer agents. Curr. Med. Chem. 18 (4), 523–538. 10.2174/092986711794480221 21143124

[B171] Shen S. Tong Y. Luo Y. Huang L. Gao W. (2022). Biosynthesis, total synthesis, and pharmacological activities of aryltetralin-type lignan podophyllotoxin and its derivatives. Nat. Product. Rep. 39 (9), 1856–1875. 10.1039/d2np00028h 35913409

[B172] Shi X. Wang J. Lei Y. Cong C. Tan D. Zhou X. (2019). Research progress on the PI3K/AKT signaling pathway in gynecological cancer (review). Mol. Med. Rep. 19 (6), 4529–4535. 10.3892/mmr.2019.10121 30942405 PMC6522820

[B173] Singh S. Kamble S. N. Ramesh K. S. Fulzele D. P. (2020). Heterologous overexpression of Nothapodytes foetida strictosidine synthase enhances levels of anti-cancer compound camptothecin in Ophiorrhiza rugosa. Plant Cell, Tissue Organ Cult. (PCTOC) 141 (1), 67–76. 10.1007/s11240-020-01767-9

[B174] Singla R. K. Sharma P. Ankit Kumar D. Gundamaraju R. Kumar D. Kumar S. (2021). Natural product-based studies for the management of castration-resistant prostate cancer: Computational to clinical studies. Front. Pharmacol. 12, 732266. 10.3389/fphar.2021.732266 34737700 PMC8560712

[B175] Sirikantaramas S. Yamazaki M. Saito K. (2013). Chapter five – camptothecin: Biosynthesis, biotechnological production and resistance mechanism(S). Adv. Botanical Res. 68, 139–161. 10.1016/B978-0-12-408061-4.00005-5

[B176] Skeel R. T. Khleif S. N. (2011). “Handbook of cancer chemotherapy,” in A lippincott Williams \& wilkins handbook. Wolters kluwer/lippincott Williams \& wilkins health. Available at: https://books.google.co.in/books?id=6Nz%5C_87OLrtcC .

[B177] Škubník J. Vladimíra Svobodová P. Ruml T. Rimpelová S. (2021). Vincristine in combination therapy of cancer: Emerging trends in clinics. Biology 10 (9), 849. 10.3390/biology10090849 34571726 PMC8468923

[B178] Srivastava A. Raghuwanshi R. (2021). “10 – landscape of natural product diversity in land-plants as source for anticancer molecules,” in Evolutionary diversity as a source for anticancer molecules. Editors Srivastava A. K. Kumar Kannaujiya V. Singh R. K. Divya S. (Academic Press), 233–254. 10.1016/B978-0-12-821710-8.00010-2

[B179] Sung H. Ferlay J. Siegel R. L. Laversanne M. Soerjomataram I. Jemal A. (2021). Global cancer statistics 2020: GLOBOCAN estimates of incidence and mortality worldwide for 36 cancers in 185 countries. CA A Cancer J. Clin. 71 (3), 209–249. 10.3322/CAAC.21660 33538338

[B180] Swamy M. K. Das T. Nandy S. Mukherjee A. Pandey D. K. Dey A. (2022). “8 – endophytes for the production of anticancer drug, paclitaxel,” in Paclitaxel. Editors Mallappa Kumara S. Pullaiah T. Zhe-Sheng C. (Academic Press), 203–228. 10.1016/B978-0-323-90951-8.00012-6

[B181] Swamy M. K. Uma Rani S. Ali G. (2018). Anticancer potential of rosmarinic acid and its improved production through biotechnological interventions and functional genomics. Appl. Microbiol. Biotechnol. 102 (18), 7775–7793. 10.1007/s00253-018-9223-y 30022261

[B182] Sztiller-Sikorska M. Czyz M. (2020). Parthenolide as cooperating agent for anti-cancer treatment of various malignancies. Pharmaceuticals 13 (8), 194. 10.3390/ph13080194 32823992 PMC7466132

[B183] Thomson C. A. Ho E. Strom M. B. (2016). Chemopreventive properties of 3,3’-diindolylmethane in breast cancer: Evidence from experimental and human studies. Nutr. Rev. 74 (7), 432–443. 10.1093/nutrit/nuw010 27261275 PMC5059820

[B184] Tian B. Liu J. (2020). Resveratrol: A review of plant sources, synthesis, stability, modification and food application. J. Sci. Food Agric. 100 (4), 1392–1404. 10.1002/jsfa.10152 31756276

[B185] Tomasetti C. Marchionni L. Nowak M. A. Parmigiani G. Vogelstein B. (2015). Only three driver gene mutations are required for the development of lung and colorectal cancers. Proc. Natl. Acad. Sci. 112 (1), 118–123. 10.1073/pnas.1421839112 25535351 PMC4291633

[B186] Tomeh M. A. Hadianamrei R. Zhao X. (2019). A review of curcumin and its derivatives as anticancer agents. Int. J. Mol. Sci. 20 (5), 1033. 10.3390/ijms20051033 30818786 PMC6429287

[B187] Tong L. Li J. Li Q. Wang X. Medikonda R. Zhao T. (2020). ACT001 reduces the expression of PD-L1 by inhibiting the phosphorylation of STAT3 in glioblastoma. Theranostics 10 (13), 5943–5956. 10.7150/thno.41498 32483429 PMC7254983

[B188] Tourlaki A. Germiniasi F. Rossi L. C. Veraldi S. Brambilla L. (2020). Paclitaxel as first- or second-line treatment for HIV-negative kaposi’s sarcoma: A retrospective study of 58 patients. J. Dermatological Treat. 31 (2), 183–185. 10.1080/09546634.2019.1590520 30897011

[B189] Umar H. I. Isaac Olatunde A. Segun Micheal A. Festus O. I. Siraj B. (2021). *In silico* molecular docking of bioactive molecules isolated from Raphia taedigera seed oil as potential anti-cancer agents targeting vascular endothelial growth factor receptor-2. Chem. Afr. 4 (1), 161–174. 10.1007/s42250-020-00206-8

[B190] Varan G. Varan C. Süleyman Can Ö. JuanBenito M. Esendağlı G. Bilensoy E. (2021). Therapeutic efficacy and biodistribution of paclitaxel-bound amphiphilic cyclodextrin nanoparticles: Analyses in 3D tumor culture and tumor-bearing animals *in vivo* . Nanomaterials 11 (2), 515. 10.3390/nano11020515 33670527 PMC7922126

[B191] Venturelli S. Burkard M. Martin B. Lauer U. M. Frank J. Busch C. (2016). Prenylated chalcones and flavonoids for the prevention and treatment of cancer. Nutrition 32 (11), 1171–1178. 10.1016/j.nut.2016.03.020 27238957

[B192] Verma V. Sharma S. Gaur K. Kumar N. (2022). Role of vinca alkaloids and their derivatives in cancer therapy.

[B193] Wang C-Z. Li B. Wen X-D. Zhang Z. Yu C. Tyler D. C. (2013). Paraptosis and NF-?b activation are associated with protopanaxadiol-induced cancer chemoprevention. BMC Complementary Altern. Med. 13 (1), 2. 10.1186/1472-6882-13-2 PMC3575249 23281928

[B194] Wang D. Neupane P. Ragnarsson L. Capon R. J. Lewis R. J. (2022). Diindolylmethane derivatives: New selective blockers for T-type calcium channels. Membranes 12 (8), 749. 10.3390/membranes12080749 36005664 PMC9412534

[B195] Wang S. Q. Cheng L-S. Liu Y. Wang J-Y. Jiang W. (2016). Indole-3-Carbinol (I3C) and its major derivatives: Their pharmacokinetics and important roles in hepatic protection. Curr. Drug Metab. 17 (4), 401–409. 10.2174/1389200217666151210125105 26651978

[B196] Wang Y. Xiao M. Sun J. Lu C. (2016). “Chapter 6 – oxidative stress in diabetes: Molecular basis for diet supplementation,” in Molecular nutrition and diabetes. Editor Didac M. (San Diego: Academic Press), 65–72. 10.1016/B978-0-12-801585-8.00006-3

[B197] Weaver B. A. (2014). How taxol/paclitaxel kills cancer cells. Mol. Biol. Cell 25 (18), 2677–2681. 10.1091/mbc.e14-04-0916 25213191 PMC4161504

[B198] Wei J. Chen F. Liu Y. Abudoukerimu A. Zheng Q. Zhang X. (2020). Comparative metabolomics revealed the potential antitumor characteristics of four endophytic fungi of Brassica rapa L. ACS Omega 5 (11), 5939–5950. 10.1021/acsomega.9b04258 32226874 PMC7098042

[B199] Whitlock N. C. Baek S. J. (2012). The anticancer effects of resveratrol: Modulation of transcription factors. Nutr. Cancer 64 (4), 493–502. 10.1080/01635581.2012.667862 22482424 PMC3349800

[B200] Willenbacher E. Khan S. Z. Mujica S. C. A. Trapani D. Hussain S. Wolf D. (2019). Curcumin: New insights into an ancient ingredient against cancer. Int. J. Mol. Sci. 20 (8), 1808. 10.3390/ijms20081808 31013694 PMC6514995

[B201] Williams D. E. (2021). Indoles derived from glucobrassicin: Cancer chemoprevention by indole-3-carbinol and 3,3’-diindolylmethane. Front. Nutr. 8, 734334. 10.3389/fnut.2021.734334 34660663 PMC8517077

[B202] Woo C. C. Loo S. Y. Gee V. Yap C. W. Sethi G. Alan Prem K. (2011). Anticancer activity of thymoquinone in breast cancer cells: Possible involvement of PPAR-γ pathway. Biochem. Pharmacol. 82 (5), 464–475. 10.1016/j.bcp.2011.05.030 21679698

[B203] Wu J. Chen W. Zhang Y. Zhang X. Jin J-M. Tang S-Y. (2020). Metabolic engineering for improved curcumin biosynthesis in Escherichia coli. J. Agric. Food Chem. 68 (39), 10772–10779. 10.1021/acs.jafc.0c04276 32864959

[B204] Xiao J. Gao M. Zhou S. Diao Q. Wang P. Gao F. (2020). Recent advances of podophyllotoxin/epipodophyllotoxin hybrids in anticancer activity, mode of action, and structure-activity relationship: An update (2010–2020). Eur. J. Med. Chem. 208, 112830. 10.1016/j.ejmech.2020.112830 32992133

[B205] Yagishita Y. Fahey J. W. Albena T. D. K. Kensler T. W. 3593 (2019). Broccoli or sulforaphane: Is it the source or dose that matters? Molecules 24 (19). 10.3390/molecules24193593 PMC6804255 31590459

[B206] Yang M. Wang H. Zhou M. Liu W. Kuang P. Liang H. (2016). The natural compound sulforaphene, as a novel anticancer reagent, targeting PI3K-AKT signaling pathway in lung cancer. Oncotarget 7 (47), 76656–76666. 10.18632/oncotarget.12307 27765931 PMC5363538

[B207] Yared J. A. Katherine H. R. T. (2012). Update on taxane development: New analogs and new formulations. Drug Des. Dev. Ther. 6, 371–384. 10.2147/DDDT.S28997 PMC3523563 23251087

[B208] Yasunaga A. Ono M. Takeshima M. Nakano S. (2022). Sulforaphane suppresses the growth of EGFR‑overexpressing MDA‑MB‑468 triple‑negative breast cancer cells *in vivo* and *in vitro* . Int. J. Funct. Nutr. 3 (2), 3. 10.3892/ijfn.2022.26

[B209] You J. S. Peter A. J. (2012). Cancer genetics and epigenetics: Two sides of the same coin? Cancer Cell 22 (1), 9–20. 10.1016/J.CCR.2012.06.008 22789535 PMC3396881

[B210] Yousefzadi M. Sharifi M. Behmanesh M. Moyano E. Bonfill M. Cusido R. M. (2010). Podophyllotoxin: Current approaches to its biotechnological production and future challenges. Eng. Life Sci. 10 (4), 281–292. 10.1002/elsc.201000027

[B211] Yu X. Che Z. Xu H. (2017). Recent advances in the chemistry and biology of podophyllotoxins. Chem. – A Eur. J. 23 (19), 4467–4526. 10.1002/chem.201602472 27726183

[B212] Zhang C. Zhang J. Wu Q. Xu B. Jin G. Qiao Y. (2019). Sulforaphene induces apoptosis and inhibits the invasion of esophageal cancer cells through MSK2/CREB/Bcl-2 and cadherin pathway *in vivo* and *in vitro* . Cancer Cell Int. 19 (1), 342. 10.1186/s12935-019-1061-1 31889894 PMC6921404

[B213] Zhang D. Kanakkanthara A. (2020). Beyond the paclitaxel and Vinca alkaloids: Next generation of plant-derived microtubule-targeting agents with potential anticancer activity. Cancers 12 (7), 1721. 10.3390/cancers12071721 32610496 PMC7407961

[B214] Zhang Xu Rakesh K. P. Shantharam C. S. Manukumar H. M. Asiri A. M. Marwani H. M. (2018). Podophyllotoxin derivatives as an excellent anticancer aspirant for future chemotherapy: A key current imminent needs. Bioorg. Med. Chem. 26 (2), 340–355. 10.1016/j.bmc.2017.11.026 29269253

[B215] Zhang Z. Li Z. Wu X. Zhang C-F. Tyler C. Tong-Chuan H. (2015). TRAIL pathway is associated with inhibition of colon cancer by protopanaxadiol. J. Pharmacol. Sci. 127 (1), 83–91. 10.1016/j.jphs.2014.11.003 25704023 PMC5053100

[B216] Zhao H. Zhu W. Jia L. Sun X. Chen G. Zhao X. (2016). Phase I study of topical epigallocatechin-3-gallate (EGCG) in patients with breast cancer receiving adjuvant radiotherapy. Br. J. Radiology 89 (1058), 20150665. 10.1259/bjr.20150665 PMC4985212 26607642

[B217] Zhao H. Zhu W. Zhao X. Li X. Zhou Z. Zheng M. (2022). Efficacy of epigallocatechin-3-gallate in preventing dermatitis in patients with breast cancer receiving postoperative radiotherapy: A double-blind, placebo-controlled, phase 2 randomized clinical trial. JAMA Dermatol. 158 (7), 779–786. 10.1001/jamadermatol.2022.1736 35648426 PMC9161122

[B218] Zhao L. Zhou J.-J. Huang X.-Y. Cheng L.-P. Pang W. Kai Z.-P. (2015). Design, synthesis and anti-proliferative effects in tumor cells of new combretastatin A-4 analogs. Chin. Chem. Lett. 26 (8), 993–999. 10.1016/j.cclet.2015.05.003

[B219] Zhao W. Cong Y. Li H-M. Li S. Shen Y. Qi Q. (2021). Challenges and potential for improving the druggability of podophyllotoxin-derived drugs in cancer chemotherapy. Nat. Prod. Rep. 38 (3), 470–488. 10.1039/D0NP00041H 32895676

[B220] Zheng J. Deng L. Chen M. Xiao X. Xiao S. Guo C. (2013). Elaboration of thorough simplified Vinca alkaloids as antimitotic agents based on pharmacophore similarity. Eur. J. Med. Chem. 65, 158–167. 10.1016/j.ejmech.2013.04.057 23708010

[B221] Zhu J. Wang M. Wen W. Yu R. (2015). Biosynthesis and regulation of terpenoid indole alkaloids in Catharanthus roseus. Pharmacogn. Rev. 9 (17), 24–28. 10.4103/0973-7847.156323 26009689 PMC4441158

[B222] Zuo S. Wang Z. Wang J. Zheng X. Xianquan A. Jing W. (2021). Self-assembly engineering nanodrugs composed of paclitaxel and curcumin for the combined treatment of triple negative breast cancer. Front. Bioeng. Biotechnol. 9, 747637. 10.3389/fbioe.2021.747637 34504835 PMC8421550

[B223] Żwawiak J. Zaprutko L. (2014). A brief history of taxol. J. Med. Sci. 1, 47–52. 10.20883/medical.e43

