# Supplementary figures and images for: Biosynthesis of anticancer phytochemical compounds and their chemistry
Source: Front Pharmacol. 2023 Mar 9;14:1136779. doi: 10.3389/fphar.2023.1136779 (PMC10034375; doi:10.3389/fphar.2023.1136779)

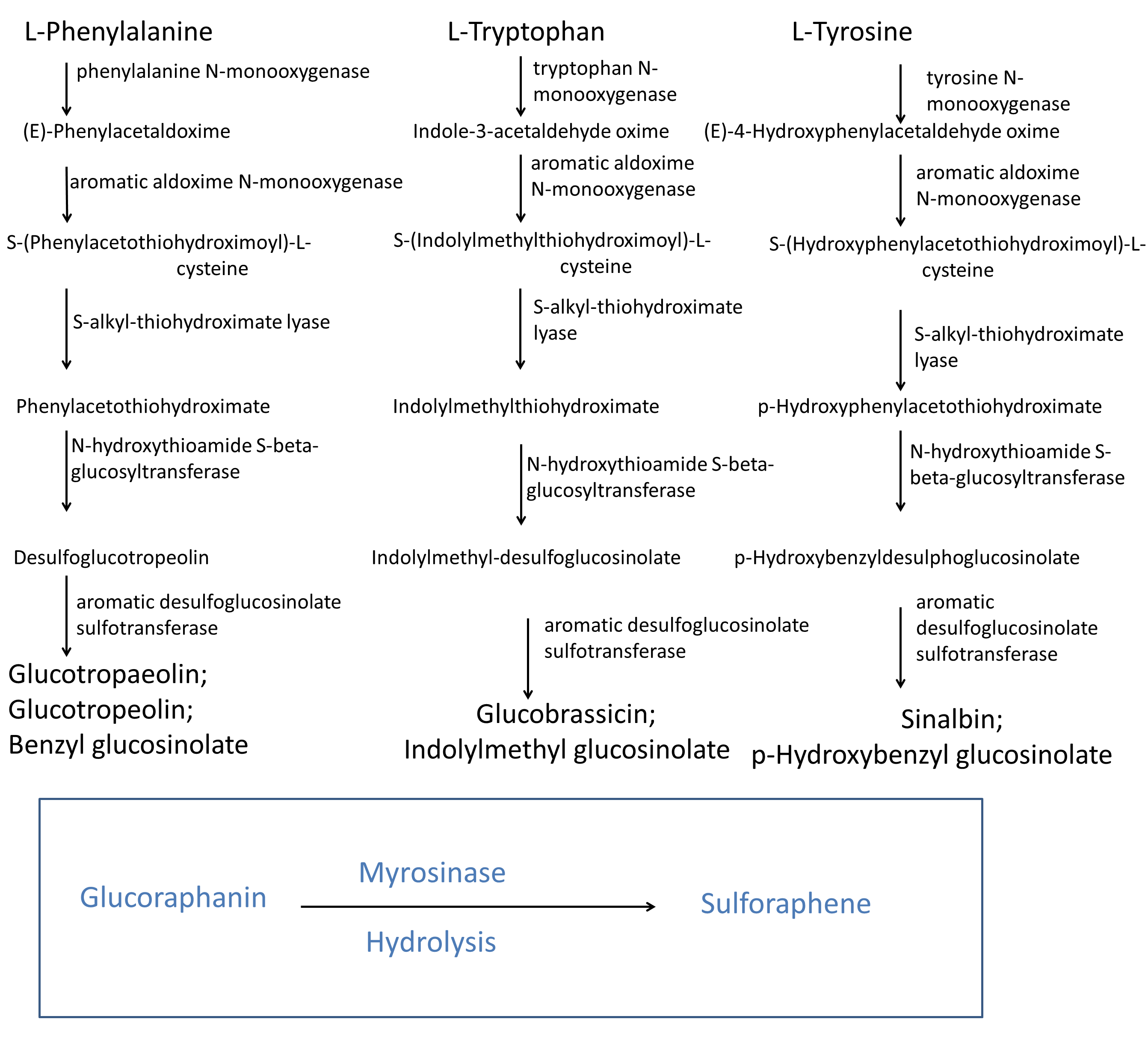

Supplement: Supplementary file 1 [file Image6.TIF]

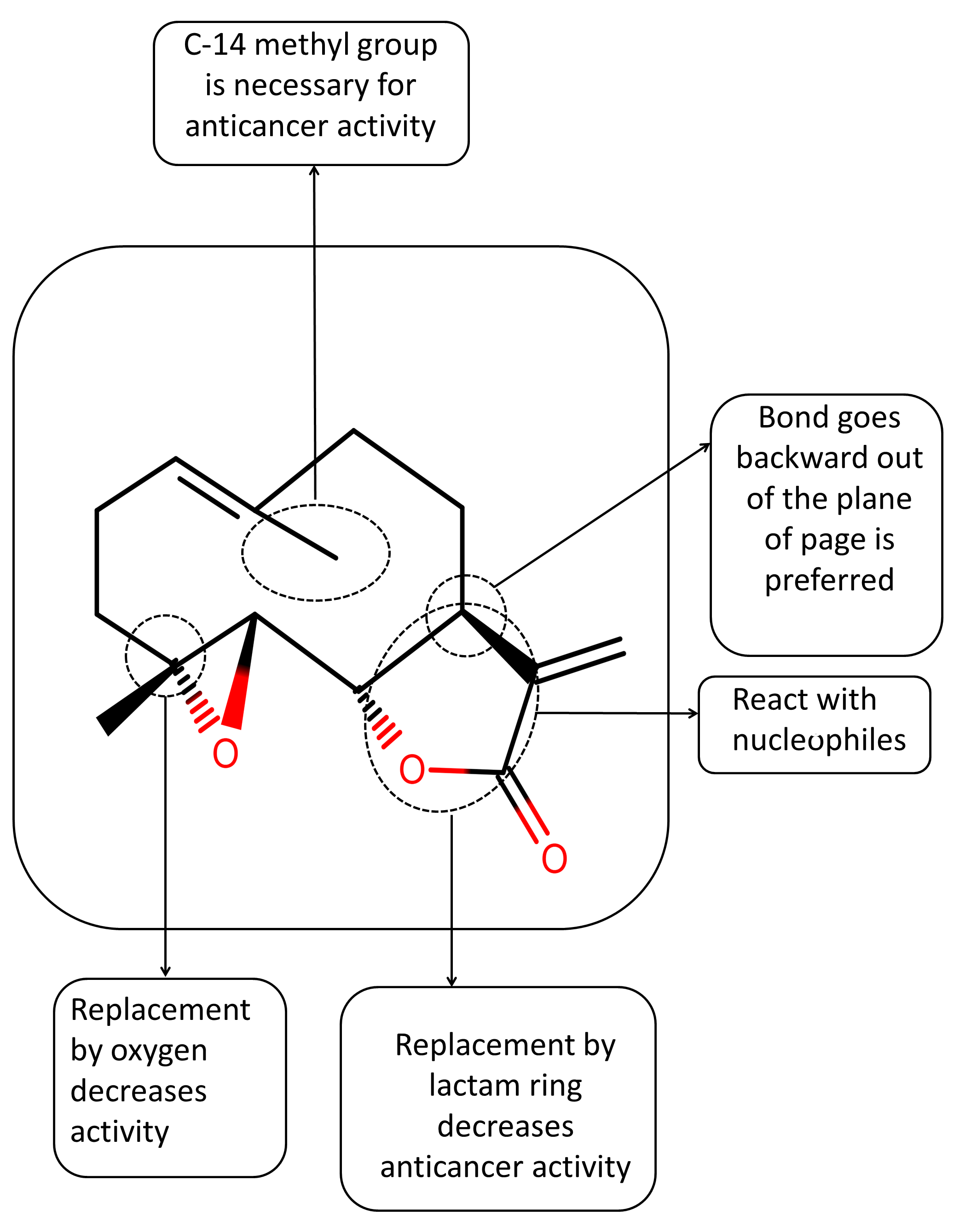

Supplement: Supplementary file 2 [file Image3.TIF]

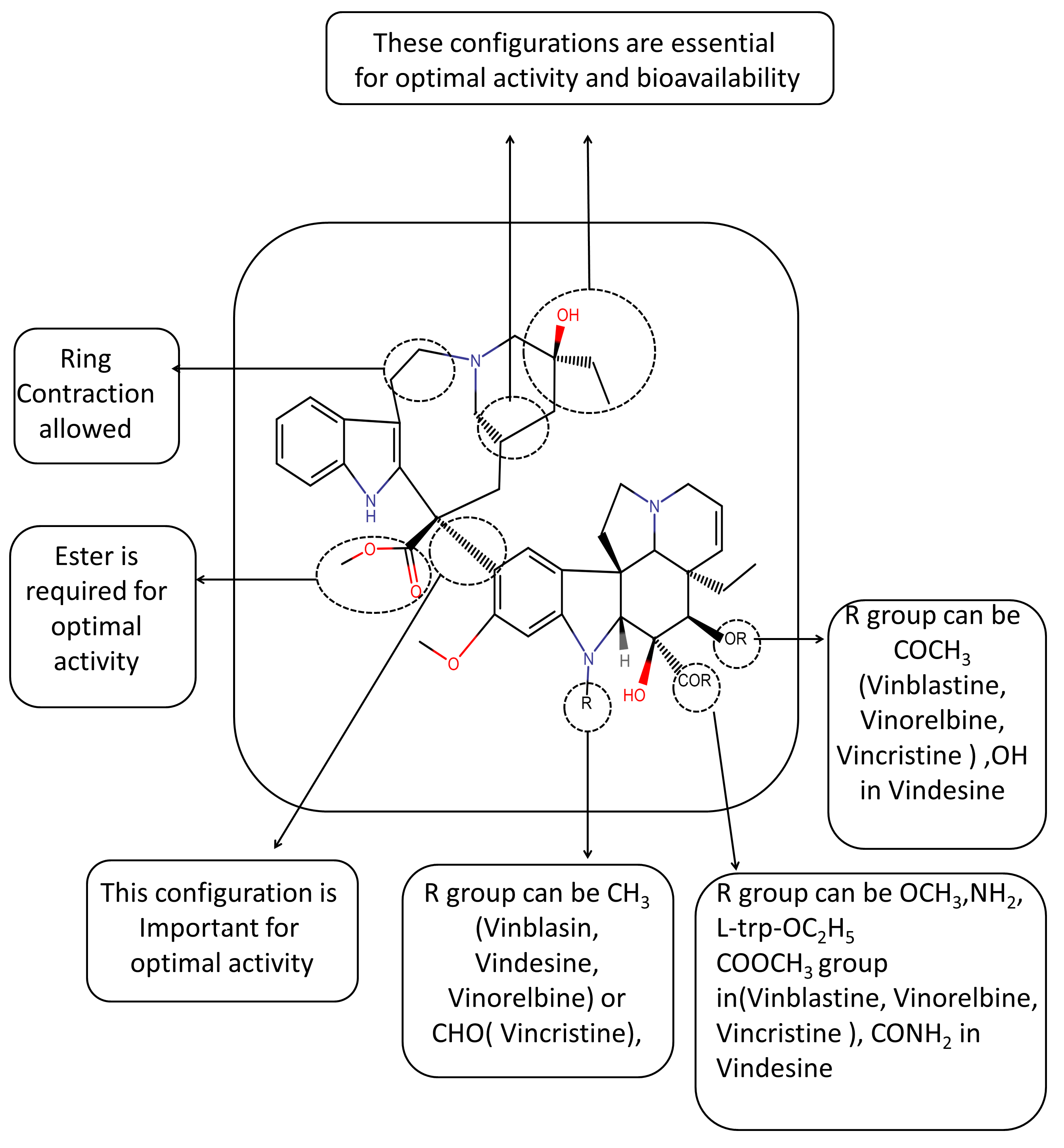

Supplement: Supplementary file 3 [file Image4.TIF]

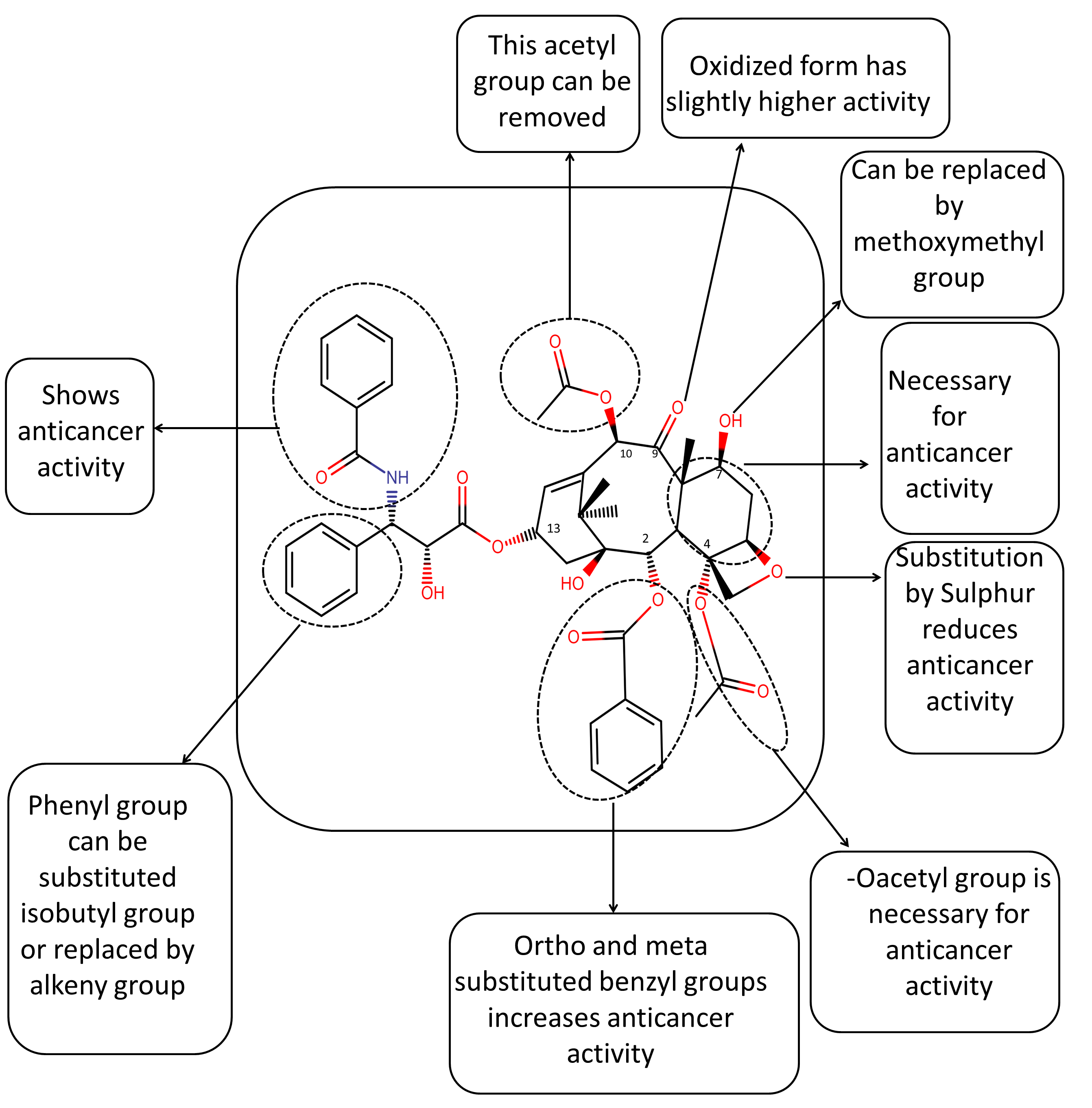

Supplement: Supplementary file 4 [file Image2.TIF]

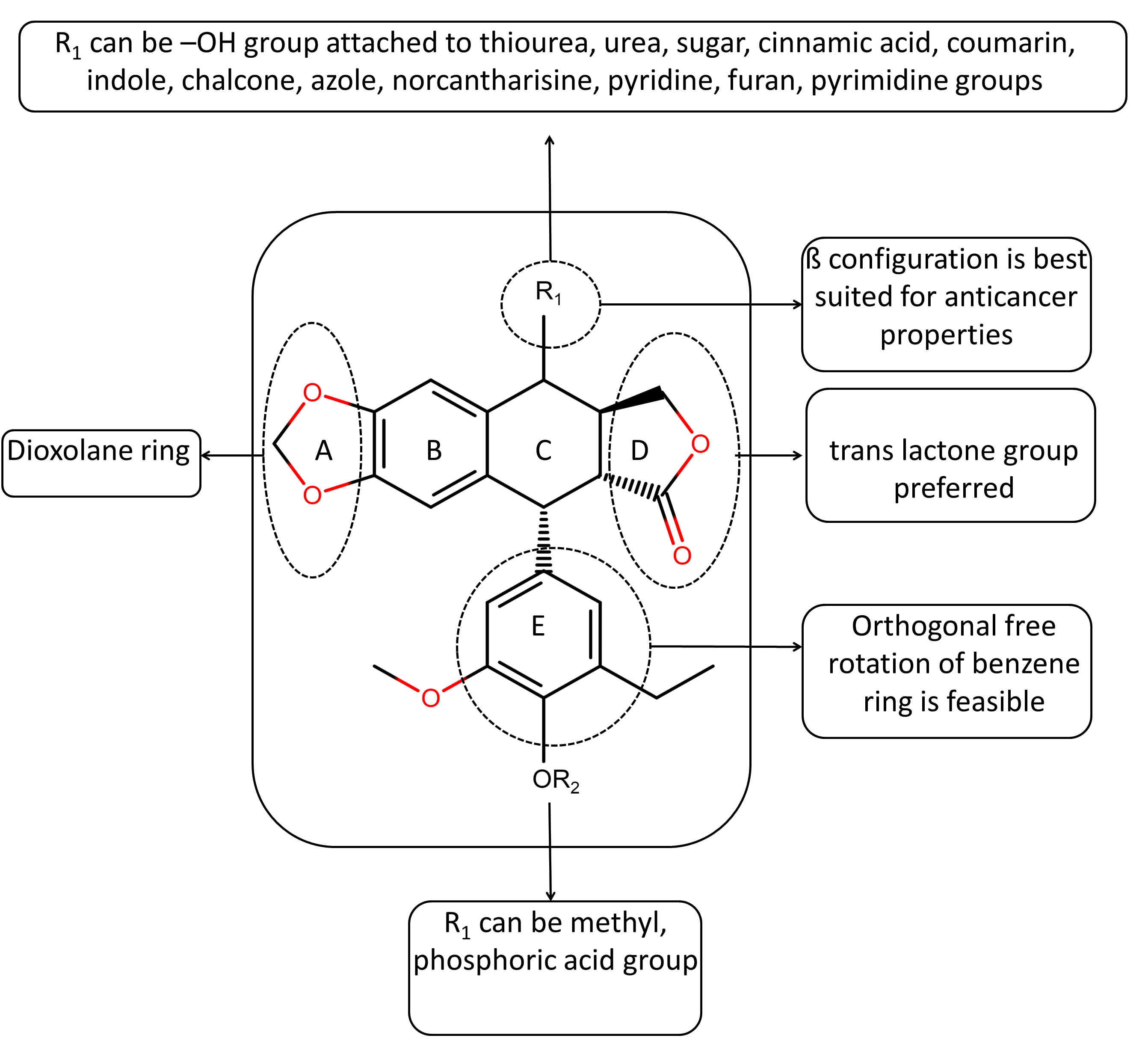

Supplement: Supplementary file 5 [file Image1.TIF]

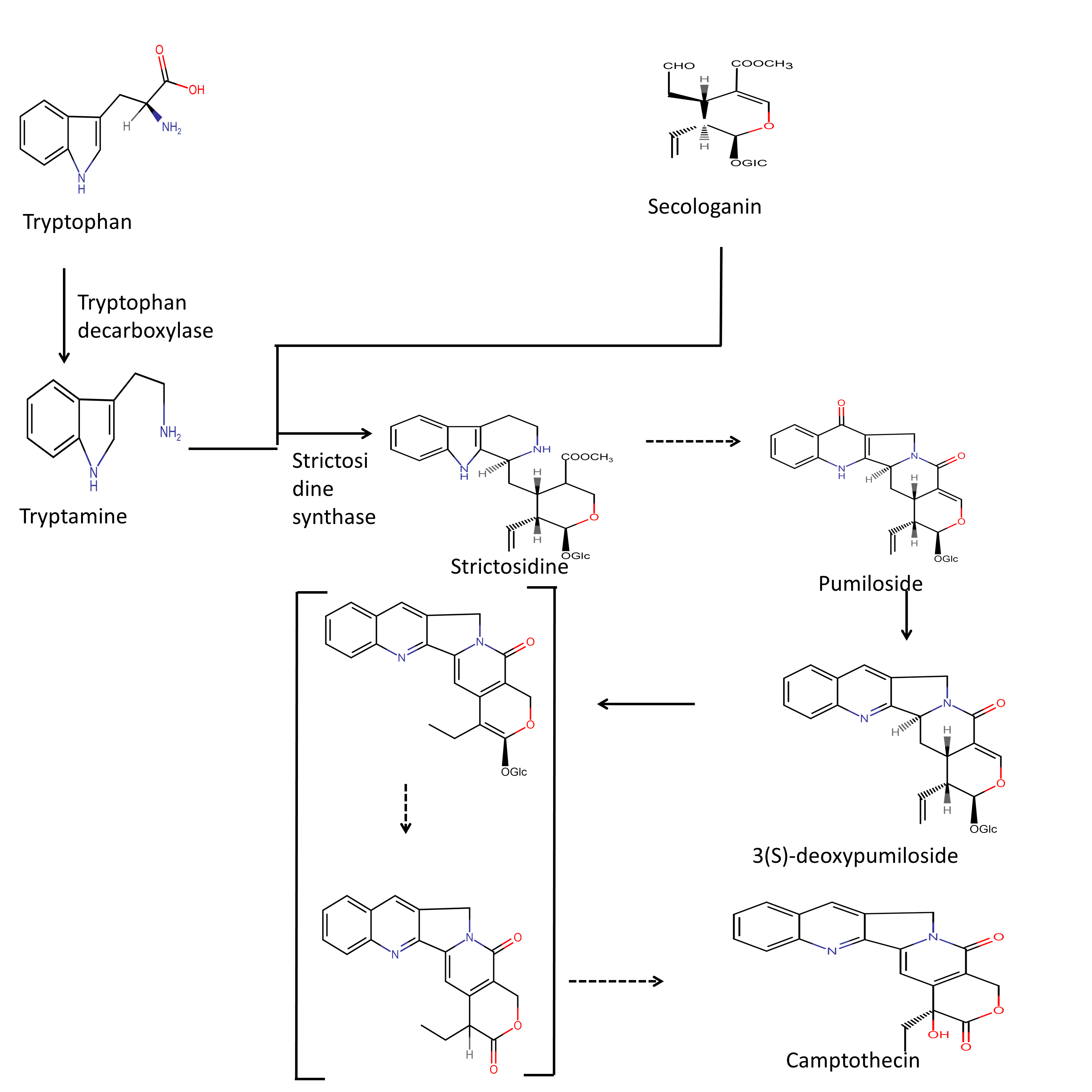

Supplement: Supplementary file 6 [file Image7.TIF]

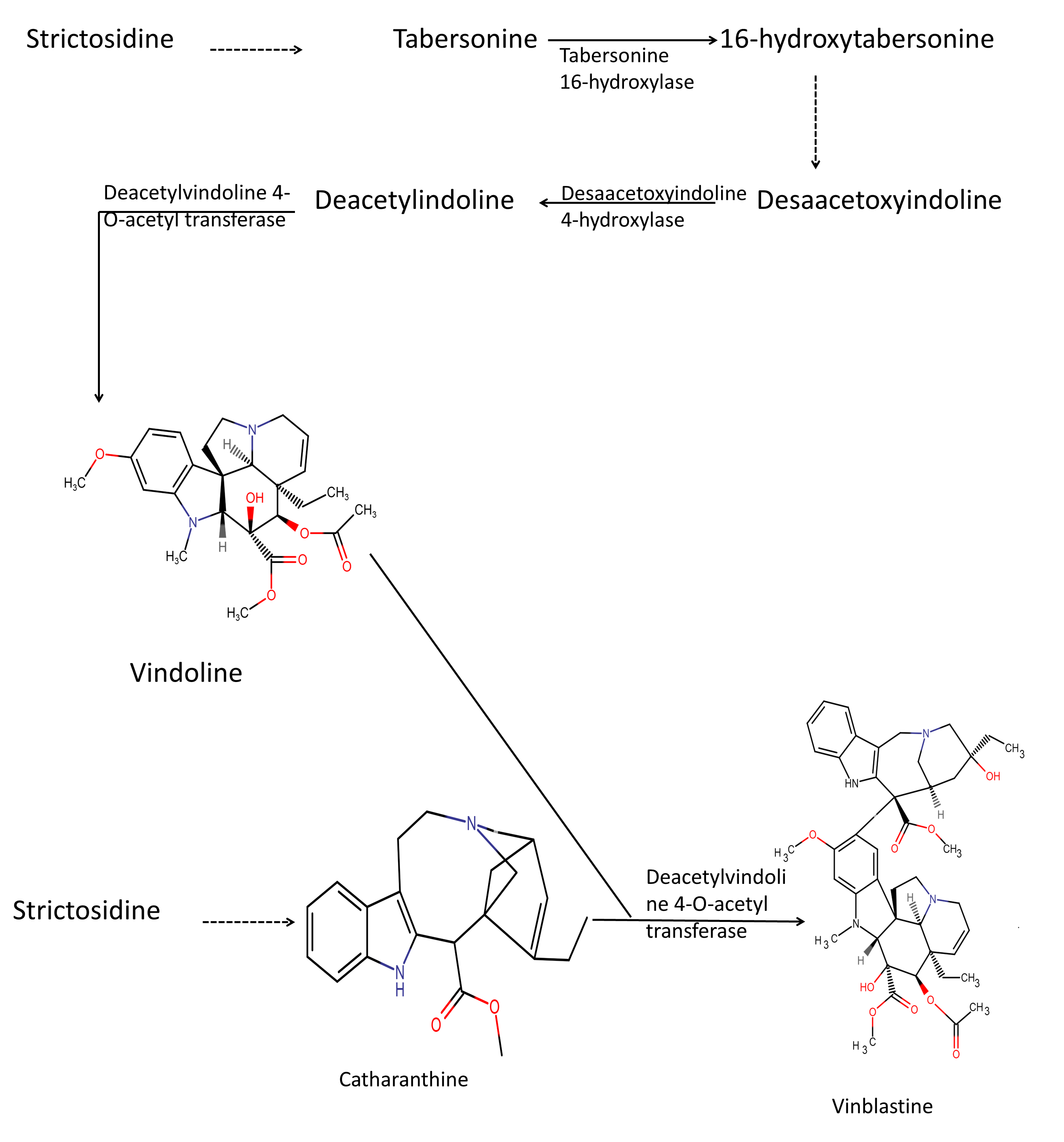

Supplement: Supplementary file 7 [file Image8.TIF]

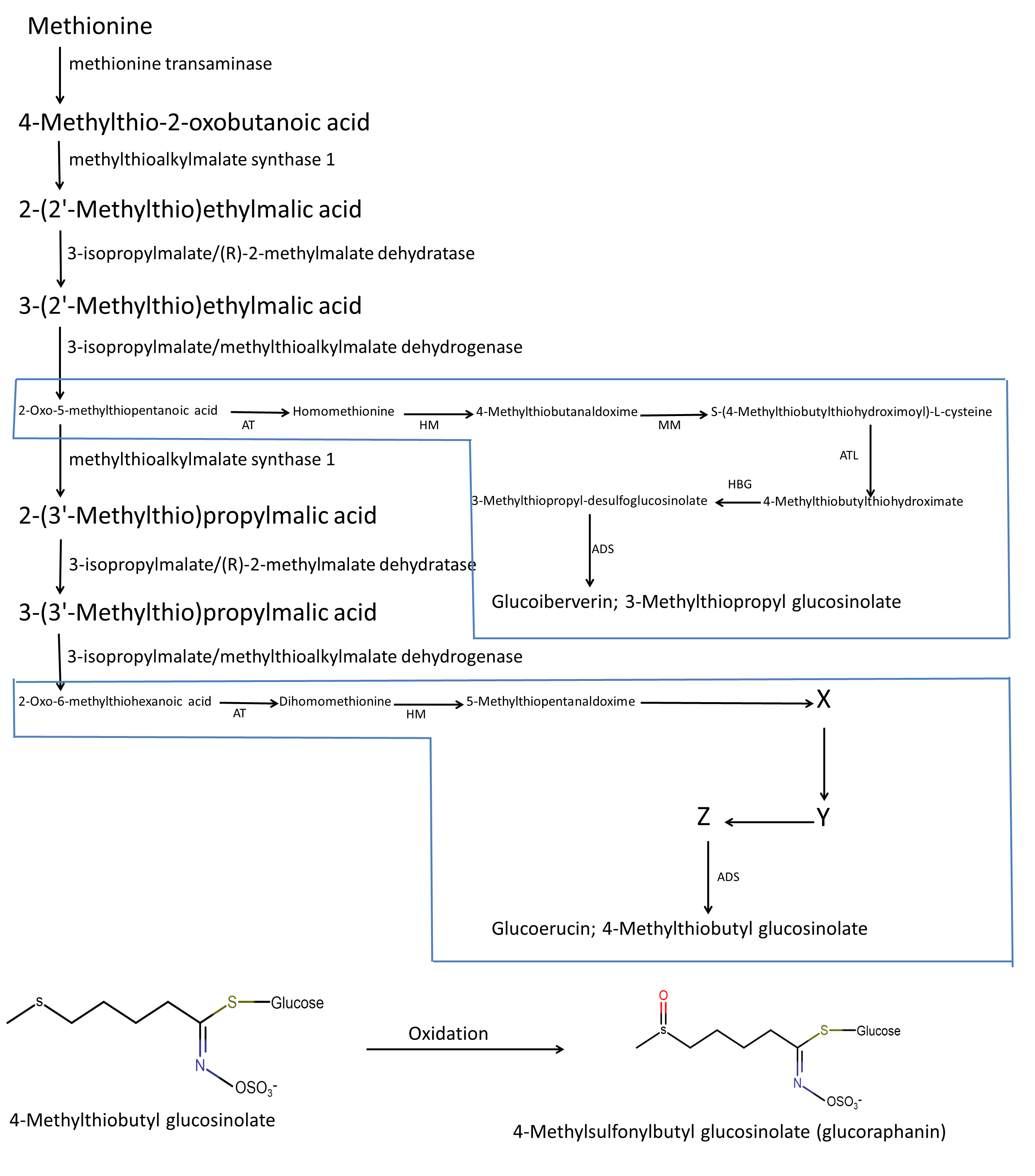

Supplement: Supplementary file 8 [file Image5.TIF]
